# A first characterization of the microbiota-resilience link in swine

**DOI:** 10.1186/s40168-024-01771-7

**Published:** 2024-03-15

**Authors:** Enrico Mancin, Christian Maltecca, Yi Jian Huang, Roberto Mantovani, Francesco Tiezzi

**Affiliations:** 1 Department of Agronomy, Animals and Environment, (DAFNAE), Food, Natural Resources, University of Padova, Viale del Università 14, 35020 Legnaro (Padova), Italy; 2 Department of Animal Science, North Carolina State University, Raleigh, NC 27695 USA; 3 Department of Agriculture, Food, Environment and Forestry (DAGRI), University of Florence, Piazzale delle Cascine 18, 50144 Firenze, Italy; 4 Smithfield Premium Genetics, Rose Hill, NC 28458 USA

**Keywords:** Gut microbiota, Pigs, Resilience, Animal-health

## Abstract

**Background:**

The gut microbiome plays a crucial role in understanding complex biological mechanisms, including host resilience to stressors. Investigating the microbiota-resilience link in animals and plants holds relevance in addressing challenges like adaptation of agricultural species to a warming environment. This study aims to characterize the microbiota-resilience connection in swine. As resilience is not directly observable, we estimated it using four distinct indicators based on daily feed consumption variability, assuming animals with greater intake variation may face challenges in maintaining stable physiological status. These indicators were analyzed both as linear and categorical variables. In our first set of analyses, we explored the microbiota-resilience link using PERMANOVA, α-diversity analysis, and discriminant analysis. Additionally, we quantified the ratio of estimated microbiota variance to total phenotypic variance (microbiability). Finally, we conducted a Partial Least Squares-Discriminant Analysis (PLS-DA) to assess the classification performance of the microbiota with indicators expressed in classes.

**Results:**

This study offers four key insights. Firstly, among all indicators, two effectively captured resilience. Secondly, our analyses revealed robust relationship between microbial composition and resilience in terms of both composition and richness. We found decreased α-diversity in less-resilient animals, while specific amplicon sequence variants (ASVs) and KEGG pathways associated with inflammatory responses were negatively linked to resilience. Thirdly, considering resilience indicators in classes, we observed significant differences in microbial composition primarily in animals with lower resilience. Lastly, our study indicates that gut microbial composition can serve as a reliable biomarker for distinguishing individuals with lower resilience.

**Conclusion:**

Our comprehensive analyses have highlighted the host-microbiota and resilience connection, contributing valuable insights to the existing scientific knowledge. The practical implications of PLS-DA and microbiability results are noteworthy. PLS-DA suggests that host-microbiota interactions could be utilized as biomarkers for monitoring resilience. Furthermore, the microbiability findings show that leveraging host-microbiota insights may improve the identification of resilient animals, supporting their adaptive capacity in response to changing environmental conditions. These practical implications offer promising avenues for enhancing animal well-being and adaptation strategies in the context of environmental challenges faced by livestock populations.

Video Abstract

**Supplementary Information:**

The online version contains supplementary material available at 10.1186/s40168-024-01771-7.

## Background

Resilience, as per the definition proposed by Holling [1], refers to a system’s ability to maintain or rapidly recover from adverse events. In the context of biological systems, it signifies the system’s capacity to cope with external disturbances [2].

Recently, resilience has gained significant attention in animal and plant science [3, 4]. Understanding the complex biological processes that govern resilience is not only scientifically attracting but also highly relevant for addressing emerging challenges, such as developing agricultural methods capable of adapting to climate change [5].

In the livestock sector, resilience is an active area of investigation, particularly in the face of the numerous challenges stemming from high-productivity systems [6, 7]. These challenges include concerns about animal welfare, the emergence of antibiotic-resistant bacteria posing risks to human health, disease outbreaks, and subsequent economic losses [8]. Consequently, identifying the mechanisms that regulate animal resilience is crucial for fostering robust, healthy, and productive livestock [9]. Additionally, exploring resilience in species such as pigs, which serve as a valuable model organism, can provide significant insights and enhance our understanding when analyzing plasticity in human physiological functions and diseases response [10].

The recent emphasis on precision farming in the livestock sector has been bolstered by advancements in high-throughput phenotyping technologies [11]. These emerging technologies, such as computerized feed intake record systems [12], have confirmed their value in identifying and studying novel phenotypes, including resilience [13]. Since resilience is tightly linked to an animal’s response to various stimuli, these technologies represent a promising opportunity to capture such responses more effectively through continuous monitoring.

In recent years, these advancements in technology have expanded the range of measurable phenotypes that can be used to assess resilience, based on the within-animal variability in such phenotype [14, 15]. One particular phenotype that has gained significant attention is feed intake [16]. In fact, studies have demonstrated a clear connection between anomalies in feed consumption (i.e., deviations from normal feeding behavior) and underlying stressors or diseases [17] in both human and livestock populations [13].

Owing to what stated above, multiple studies have been undertaken to elucidate the genetic mechanisms responsible for resilience. These investigations have employed diverse approaches, ranging from high-throughput phenotypic technologies [13, 16] to conventional data collection systems [18, 19]. All of these studies underscore how it exists a direct genetic control of resilience. Particularly, a complex set of genes, seemingly associated with the immunity system [12, 13, 17], appear to play a significant role. In spite of these efforts though, the precise regulatory mechanisms governing resilience remains elusive.

However, the understanding of resilience must move beyond a simplistic view that attributes its control solely to the environment and the host genome [20]. This approach may overlook important intermediate layers that exist between resilience and the host or animal’s physiology. Recent studies [21, 22] highlight one such intermediate layer by demonstrating the value of new 'omics' approaches, particularly gut metabolomics and microbiome, in identifying novel factors, such as the inflammatory response, that are linked to the animal’s resilience. In fact, research on the host-microbiota relationship has emerged as a valuable tool in elucidating eco-evolutionary processes, including resilience and its phenotypic expression [23]. In the context of animal production systems, the microbiota has expanded our knowledge of biological processes related to meat production and quality [24], feed behavior [25], and environmental impact [26]. Consequently, the microbiota holds great promise as (i) a cost-effective “marker” for indicating the biological status of animals [27] and (ii) a mean to select superior animals to achieve improved outcomes [28].

The main objective of this research is to investigate the relationship between the composition of the microbiota (i.e., the collection of microorganisms in an animal’s body) and its resilience. We aim to understand how the microbiota changes over time and between different groups of animals, and how these changes may be linked to resilience. Specifically, we will explore the connection between resilience and the richness and composition of the microbiota by (a) defining meaningful indicators of resilience in swine based on feeding data, (b) quantifying the association between microbial composition and resilience indicators, and (c) evaluating the potential of specific microbial composition to discriminate between resilient and non-resilient individuals.

By conducting this study, we hope to advance our understanding of how the microbiota influences an animal’s ability to cope with challenges and maintain its health. Ultimately, our findings may provide valuable insights and tools for managing and improving animal well-being.

## Results

### Defining indicators of resilience

This study aimed to leverage the concept of “variability” in feed consumption as an indicator of animals’ resilience. We hypothesized that animals exhibiting higher day-to-day variability in feed consumption might be more susceptible to external disturbances, including environmental and social stressors or disease, which could subsequently influence their feeding patterns. To obtain a comprehensive understanding of resilience, we investigated multiple indicators of this “variability.”

**Fig. 1:**
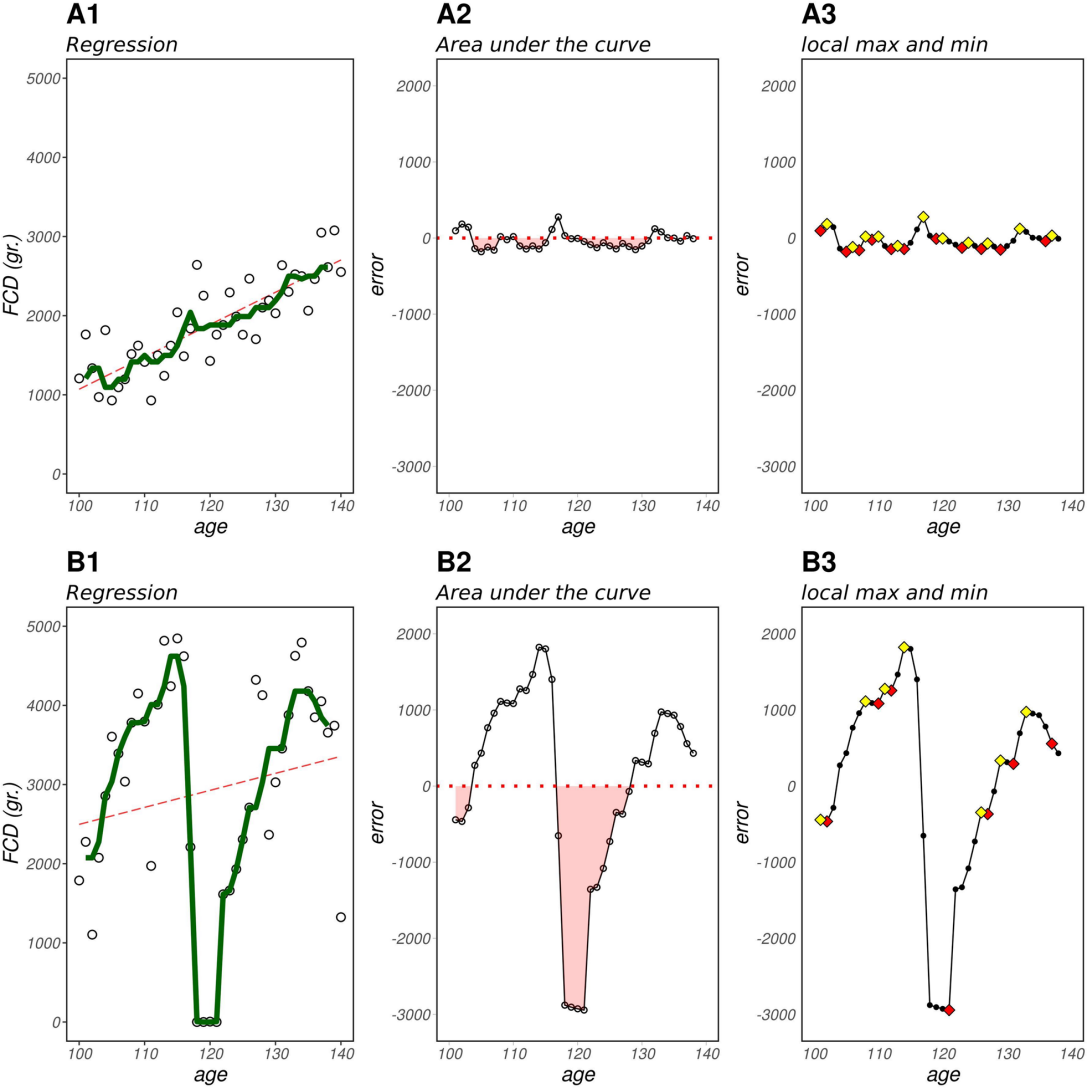
Process for obtaining the four different resilience indicators. Part A represents animals with the least variability, while Part B represents animals with higher variability. Each part includes the following representations: A1, B1 the predicted values (in red) and the observed values (in green); A2, B2 the periods of consecutive negative errors highlighted in red; A3, B3 local minima depicted in red and local maxima depicted in yellow

Figure 1 illustrates the rationale adopted to derive the various resilience indicators in this study. In order to facilitate understanding of this process, we chose to present two distinct individuals: one with higher resilience, represented by lower LnVar values (Panel A), and another with lower resilience and higher LnVar (Panel B). The first column of Figure 1 displays the observed values, depicted by the green line, which represents a moving median calculated over a 5-day period of feed consumption data (FCD). As a predictive model, linear regression was employed, and the resulting prediction is reported by the red line. Subsequently, we derived the Lag1 and LnVar indicators from the residual values (i.e., predicted values subtracted from the observed values) obtained through the regression analysis.

In the middle column of each panel (A2 and B2) of Figure 1, we illustrate the occurrence of “negative periods” in red, representing instances where there were more than two consecutive days of negative residual values, indicating when the predictor exceeded the observed values. The largest area of negative period was identified and designated as the third resilience indicator, referred to as MaxArea.

Finally, the last column (A3 and B3) of each panel highlights the local minima (in red) and local maxima (in yellow). We utilized the sum of the local minima as the last resilience indicator, labeled as SumMin.

**Fig. 2:**
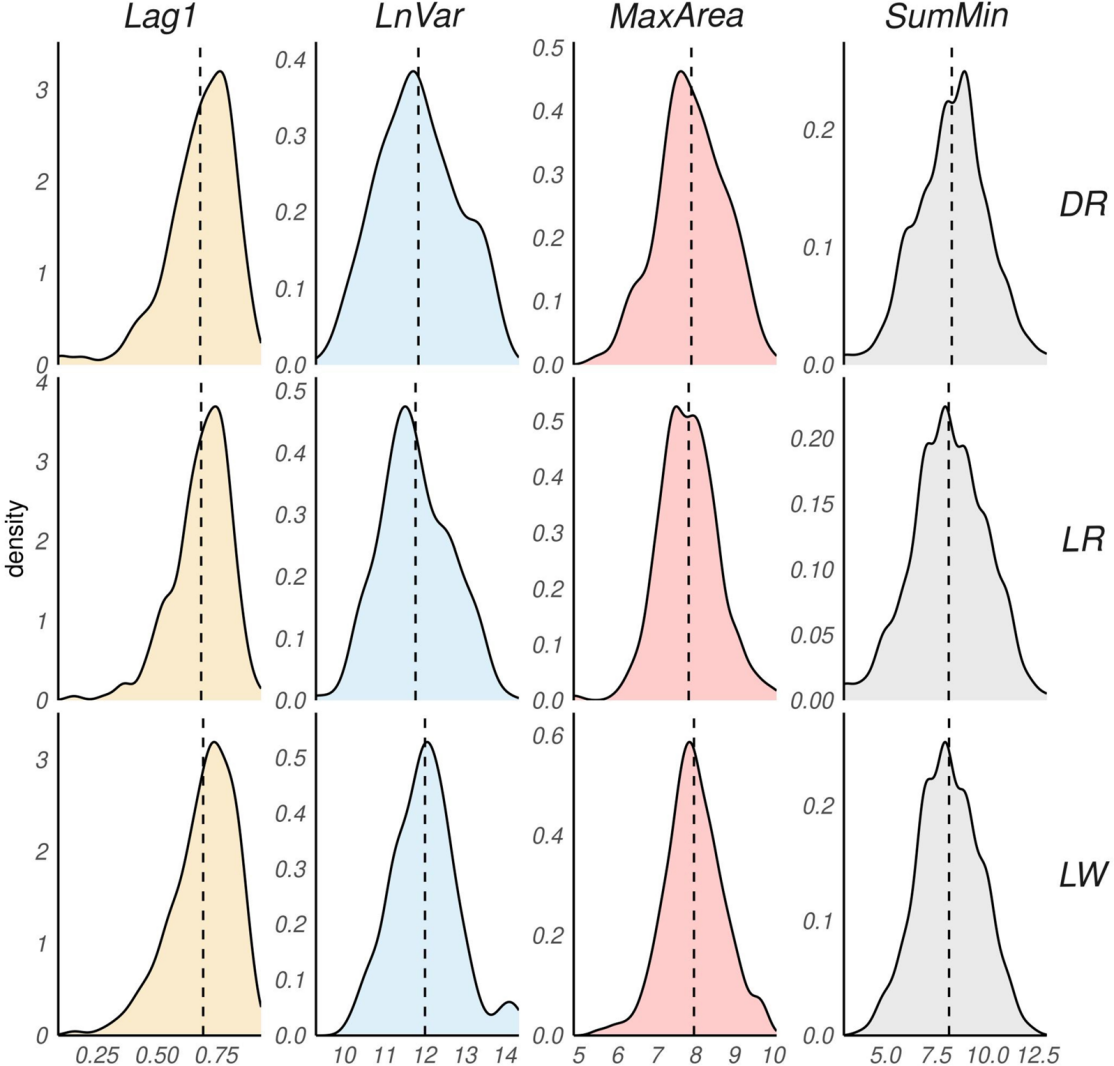
Density plot of distribution of the four different phenotypes divided by breed: Duroc (DR), Landrace (LR), Large White (LW). The dotted line represents the mean value for each breed of each phenotype: lag of 1 day of residual (Lag1), natural logarithm of residual variance (LnVar) area under the curve for periods with largest consecutive negative errors (MaxArea), sum of residual’s local minima (SumMin)

Figure 2 illustrates the distribution of the four resilience indicators. The normality assumption of these phenotypes is paramount for the subsequent analysis. In all cases, normality assumption was not violated. To investigate potential distributional differences among the breeds considered, we conducted a post hoc test using the Tukey method within a linear mixed model framework. The model incorporated effect of breed of the animal, as well as the nested effect of pen within room and the random effect of family (sire) of the animal. The results indicated that there were no significant differences observed among the breeds (*P* > 0.05). For more detailed information, please refer to Supplementary Material 1.

We performed a Spearman correlation analysis to examine the association of the resilience indicators with various performance traits of the pigs. These phenotypes included the percentage of muscle, backfat, and intramuscular fat, which were obtained at the end of the performance test, as well as the body weight measures at the end of the performance test, at an age of 156 ±2.68 days. Additionally, we incorporated two traits measured during the period in which the resilience indicators were calculated: average daily feed consumption and average daily body weight.

**Fig. 3:**
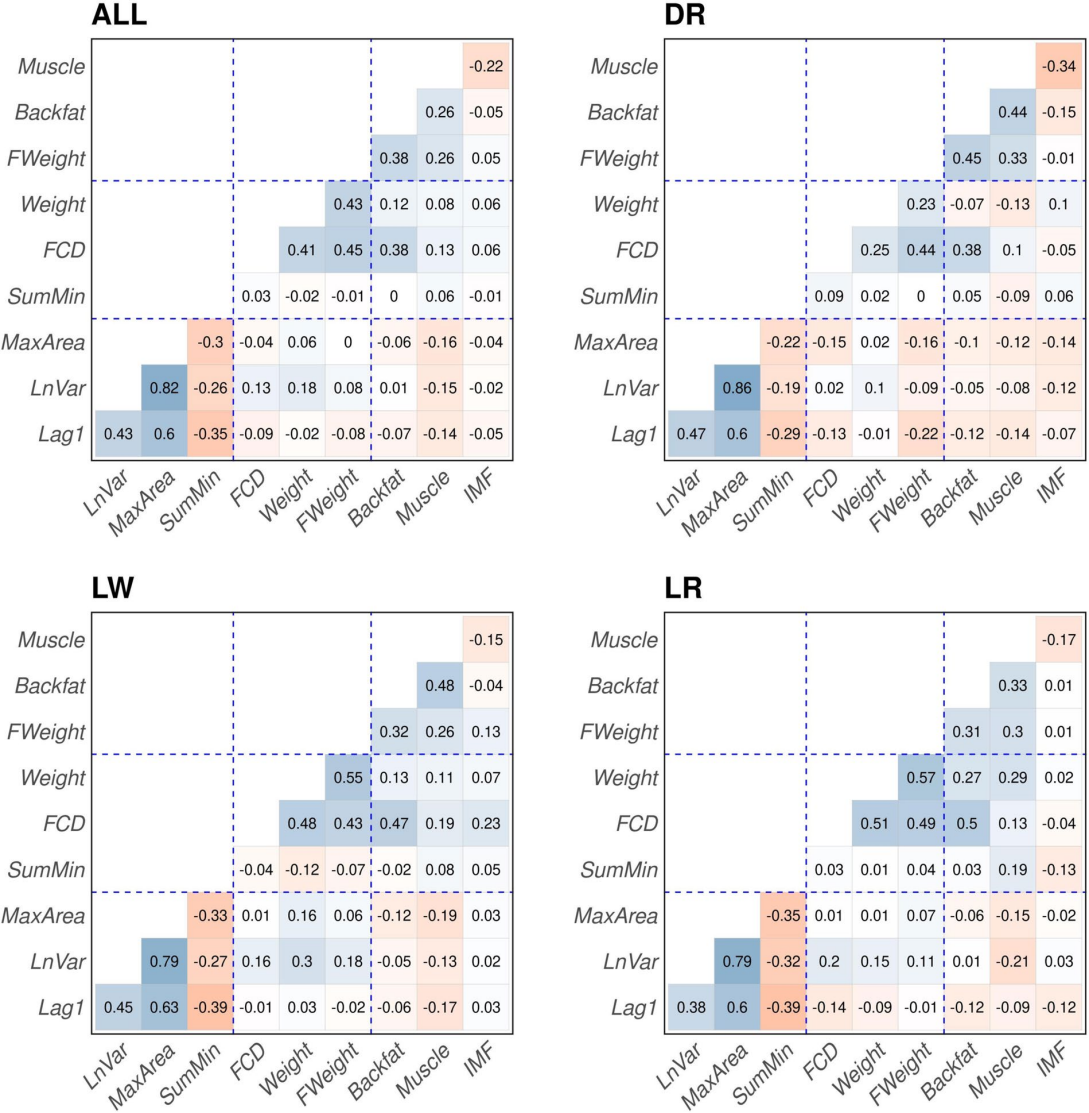
Plot representing the Spearman correlation between the four resilience indicators and other productive phenotypes. Resilience indicator were lag of 1 day of residual (Lag1), natural logarithm of residual variance (LnVar) area under the curve for periods with consecutive negative errors (MaxArea), and sum of residual’s local minima (SumMin). Productive phenotypes were average feed daily consumption (FCD) and average body weight (Weight) estimated in the period of which the four resilience indicators were estimated (99–140 days). Phenotype collected before slaughter as percentage of muscle (Muscle), and backfat and intramuscular fat (IMF) and body weight (Final_Weight). Correlation was calculated for all animals (ALL) or for animals of same breed Duroc (DR), Landrace (LR), and Large White (LW)

The Spearman correlation values estimated using all individuals are presented in the top-left pane of Figure 3 (ALL), the correlation estimates subsetting individuals from each breed Duroc (DR), Landrace (LR), and Large White (LW) are depicted in the other panels of Figure 3 (LW, LR, DR).

From the correlation between resilience indicators, a few clear patterns emerged. The Lag1, LnVar, and MaxArea exhibited positive correlations with each other, while SumMin revealed negative correlations with all other indicators, ranging from −0.32 (0.043) to −0.39 (0.04). The strongest correlation was observed between LnVar and MaxArea, with a coefficient of 0.79 (0.015). The Lag1 showed a correlation of 0.39 (0.028) with LnVar and a stronger correlation of 0.60 (0.024) with MaxArea.

When examining the correlations between resilience indicators and other productive traits, we discovered a negative correlation between the three positively correlated indicators—Lag1, LnVar, and MaxArea—and the percentage of muscle, with an average correlation coefficient of −0.15 with an average standard error of 0.04. The strongest correlation was observed between MaxArea and the percentage of muscle, with a coefficient of −0.21 (0.016). Our analysis did not reveal any significant associations between the resilience indicators and the percentage of backfat, intramuscular fat, or final body weight.

When examining the correlation between resilience indicators and traits measured during the same time window, we observed that only LnVar showed a positive correlation with average FCD and average weight. On the other hand, despite the high correlation between MaxArea and LnVar, MaxArea exhibited an almost null correlation with FCD.

The correlation results suggest that resilience indicators are traits that show low or null collinearity with traditionally recorded traits in swine production. Correlation patterns within breeds remained consistent, as observed in Figure 3 (LW, LR, DR). While the numeric values of the correlations may have varied slightly, the overall patterns remained unchanged.

### Association between microbial composition and resilience

To investigate the potential association between the microbiota and resilience, we conducted a PERMANOVA analysis to determine whether variations in the composition of the microbiota were linked to the four resilience indicators. To account for possible confounding effects, we included the effects of room and breed in the analysis, along the resilience indicators.

The results of the PERMANOVA model are presented in Table 1. We found that all resilience indicators exhibited a significant association with microbial composition. Specifically, LnVar and MaxArea had the most pronounced influence on the microbiota composition (*P* < 0.001), followed by SumMin (*P* = 0.003), and Lag1 (*P* = 0.023). It is noteworthy that, although not the primary focus of this study, we also identified a significant impact of room and breeds (*P* < 0.001), which is further detailed in Supplementary Material 2. The different indicator explained a range of 0.2% (for Lag1) to 0.6% (for LnVar and MaxArea) of the total variance. In contrast, breed and room contributed 9 and 4%, respectively, to the total microbiota variance.

**Table 1:** Results from PERMANOVA analysis for the effect each of the four resilience phenotypes on microbial composition. The four phenotypes were lag of 1 day of residual (Lag1), natural logarithm of residual variance (LnVar), area under the curve for periods with the largest consecutive negative errors (MaxArea), and sum of residual’s local minima (SumMin). Indicator was considered as linear trait

**Indicator**	**SumSq**	**F**	**Pr(>F)**	**VarExp**
Lag1	1595	1.698	0.023	0.295
LnVar	3208	3.416	<0.001	0.634
MaxArea	3408	3.629	<0.001	0.631
SumMin	2550	2.715	0.003	0.472

Indicator: Resilience phenotype; *SumSq* the total variation between the group means and the overall mean, *F test Pr(>F) P*-value of the F statistic, *VarExp* percentage of total variance explained by microbiota composition

**Fig. 4:**
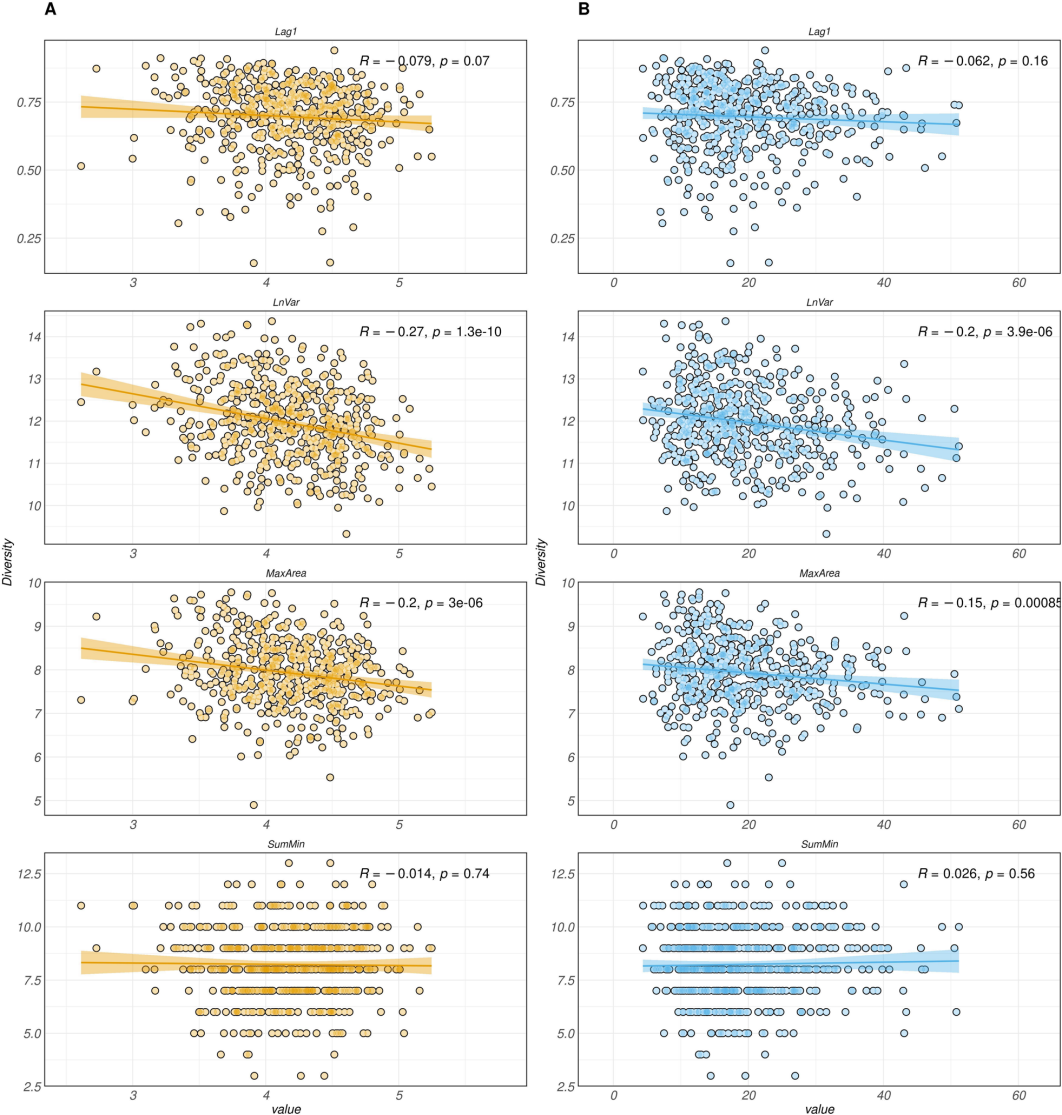
Scatter plot representing linear regression of the four resilience indicators (x-axis) on the two α-diversity measures (y-axis): Shannon (**A**) and inverse Simpson (**B**). The four resilience indicators were Lag of 1 day of residual (Lag1), natural logarithm of residual variance (LnVar) area under the curve for periods with largest consecutive negative errors (MaxArea), sum of residual’s local minima (SumMin). Coefficient of regression (R) and *P*-values (p) of each regression were reported in each plot

We investigated the association between resilience and microbiota richness by performing a regression analysis, where we used the α-diversity of bacterial communities as the predictor variable and the resilience measures as the outcome variables. To quantify microbiota richness, we employed two diversity indices: the inverse of Simpson’s index and the Shannon index. These indices capture both the richness (number of unique bacterial species) and evenness (distribution of species abundances) of the microbiota (Fig. 4).

A significant (*P* < 0.01) and consistent decrease in both α-diversity measurements was observed as LnVar and MaxArea values increased. This suggests that animals with higher variability, indicating lower resilience, exhibited reduced microbiota richness, with an R that range from −0.27 to −0.15. Similarly, negative trends were observed for Lag1, although statistical significance was not reached for the inverse Simpson index (*P* = 0.16) and the R was small (−0.079 to −0.069). However, when richness was expressed using the Shannon diversity measure, the negative trend for Lag1 was slightly above the significance threshold (*P* = 0.07). These findings further support the notion that animals with higher variability and lower resilience tend to exhibit lower microbiota richness. However, when was performed within-breed, significant link was observed only for MaxArea and LnVar (Supplementary 3).

Conversely, SumMin showed almost negligible trends, suggesting that as local minimum values increased, α-diversity remained relatively stable. Although the trends between Lag1 and microbial composition were not statistically significant, a slight decline was observed when the Shannon index was used as the richness measure.

Consequently, we aimed to identify through differential abundance (DA) analysis which ASVs and their corresponding KEGG pathways were associated with these changes in the microbiome. By identifying these ASVs, we can gain insights into the specific microbial components that contribute to the observed changes in the microbiome associated with resilience.

**Fig. 5:**
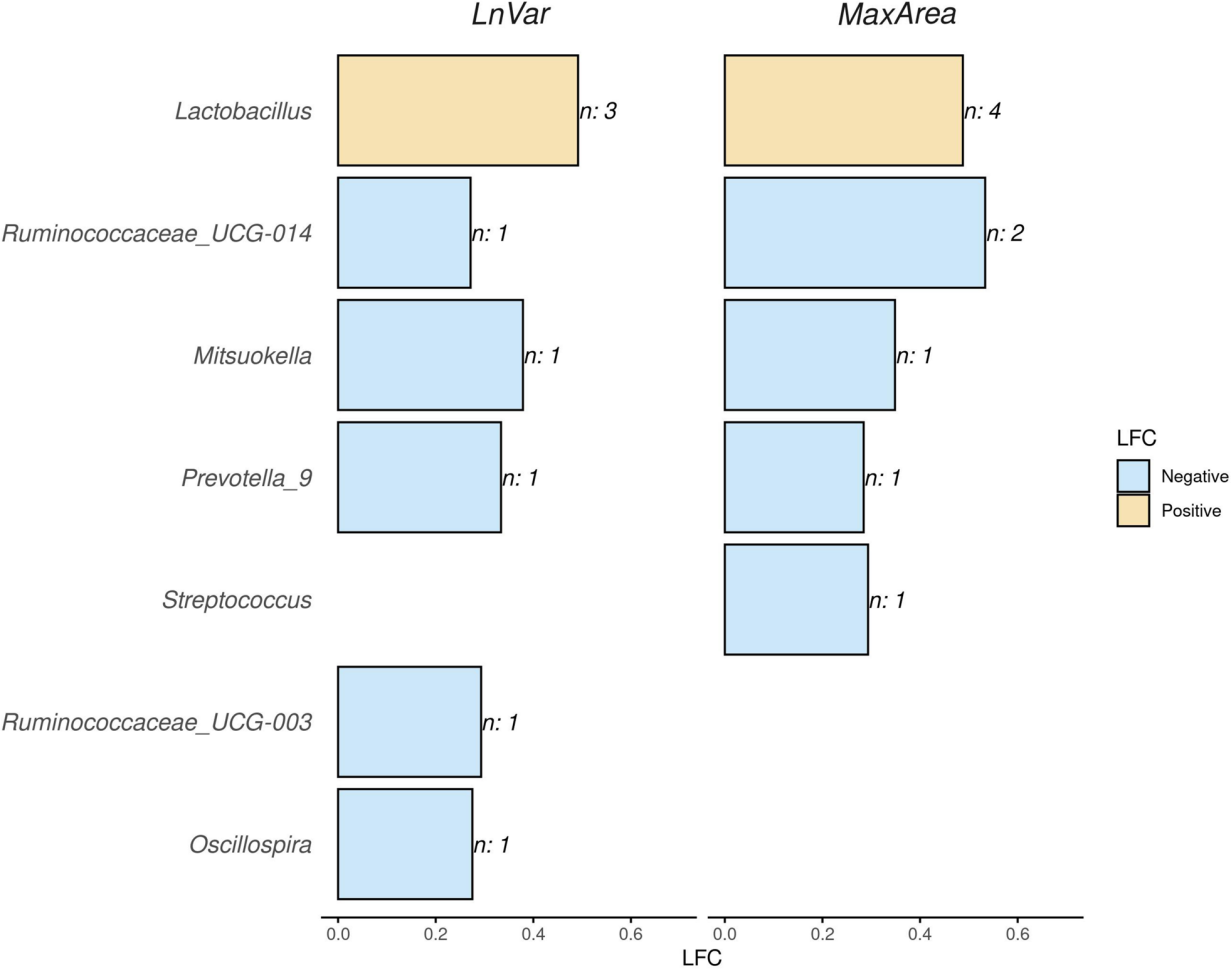
Barplot illustrating the absolute value of the log fold change (LFC) abundances for significantly abundant amplicon sequence variants (ASVs), for the indicators of natural logarithm of residual variance (LnVar) and area under the curve for periods with largest consecutive negative errors (MaxArea). ASVs with positive LFC are represented by yellow bars, while those with negative LFC are represented by light-blue bars. ASVs are grouped based on the genera to which they belong, and when multiple ASVs belong to the same genus, the mean of all ASVs within that genus is reported. The number of ASVs per genus class is indicated near each bar

Figure 5 presents the results of the ASV differential analysis. In the model, each ASV was treated as an independent factor. However, for clarity, the results in Figure 5 are aggregated at the genus level, grouping together significant ASVs belonging to the same genus. In instances where multiple ASVs were associated with the same genus, the mean Log Fold Change (LFC) of the ASVs within each genus was reported.

Among the resilience indicators, LnVar and MaxArea exhibited notable differences in ASV abundance. Specifically, LnVar showed significant variations in the abundance of 7 ASVs across 4 different genera. On the other hand, MaxArea displayed significant differences in the abundance of 10 ASVs belonging to 7 different genera. These ASVs were associated with six distinct families: *Lactobacillaceae*, *Ruminococcaceae*, *Prevotellaceae*, *Veillonellaceae*, *Lachnospiraceae*, and *Streptococcaceae*. It is important to note that the last two families were only observed in relation to the MaxArea indicator.

Regarding the genera associated with resilience indicators, *Lactobacillus* exhibited a positive LFC value of 0.46 for LnVar and 0.51 for MaxArea, indicating its higher abundance in animals with greater variability. *Lactobacillus* was represented by three different ASVs in LnVar and by four ASVs (including the three from LnVar) in MaxArea. *Rumminococcacea_UCG-014* displayed the most negative LFC values for both indicators, with −0.35 for LnVar and −0.6 for MaxArea. This genus was represented by two distinct ASVs in LnVar. Then, *Misuokella* and *Prevotella_9* exhibited LFC values of −0.34 and −0.28 for LnVar, and −0.38 and −0.33 for MaxArea, respectively. In addition, for MaxArea, ASVs belonging to the genera *Streptococcus*, *Rumminococcacea_UCG-003*, and *Oscillospira* were identified, with LFC values ranging from approximately −0.29 to −0.27.

**Fig. 6:**
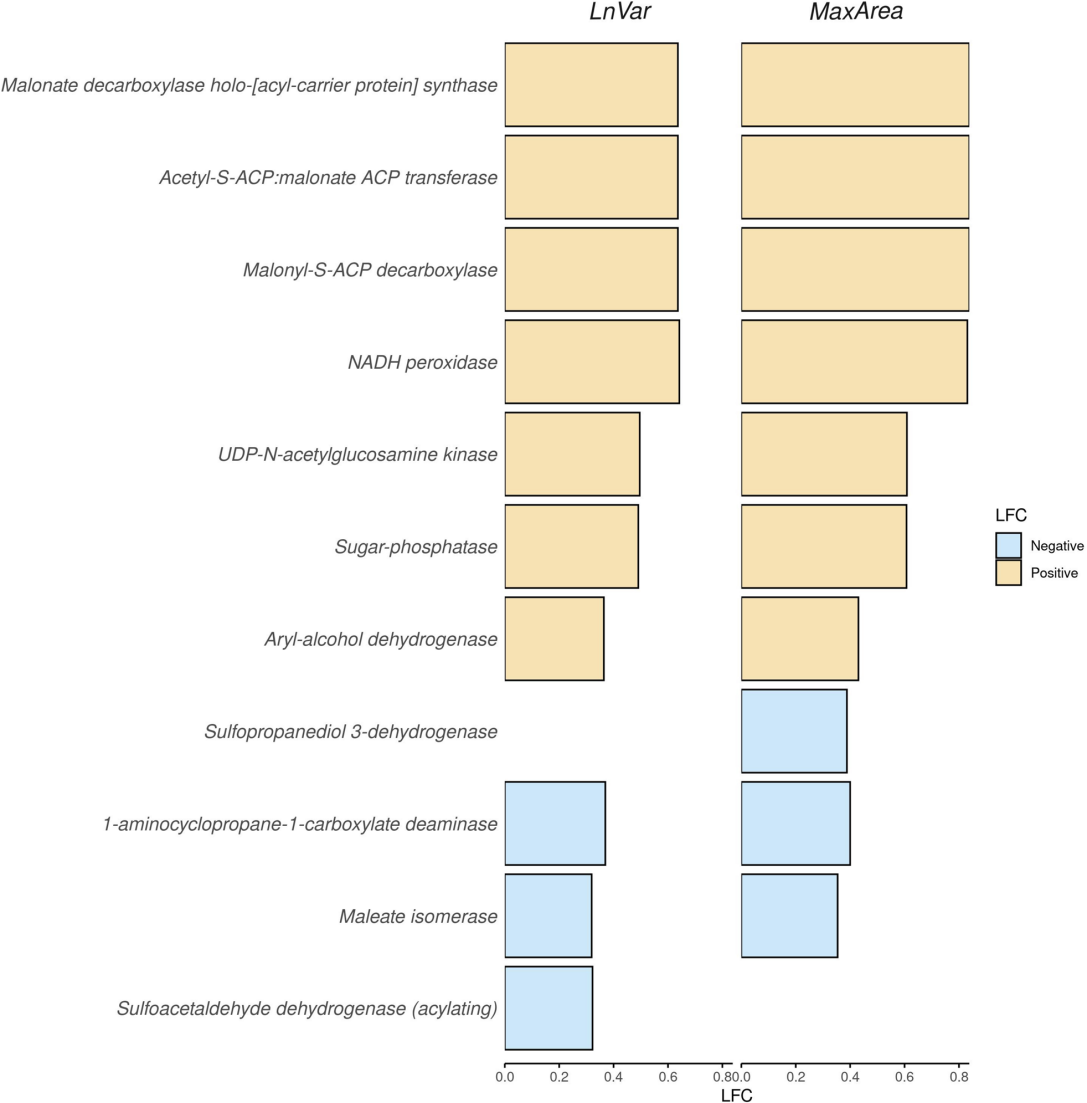
Barplot illustrating the absolute value of the log fold change (LFC) abundances for the ten most significantly abundant KEGG pathways, for the indicators of natural logarithm of residual variance (LnVar) and area under the curve for periods with consecutive negative errors (MaxArea). KEGG pathways, with positive LFC, are represented by yellow bars, while those with negative LFC are represented by light-blue bars

In addition to the analysis of individual ASVs, we also examined the abundances of KEGG pathways associated with enzymatic activities using a similar approach with the ANACOM-BC procedure. To maintain clarity and conciseness, in Figure 6, we present the top 10 most influential microbiological enzymatic activities based on the absolute values of LFC. Supplementary Material 4 includes all KEGG pathways that surpassed the threshold for corrected *P*-values (Bonferroni threshold of 0.05).

Specifically, we identified 65 different classes of enzymatic activities for LnVar and 44 for MaxArea, indicating the potential involvement of a diverse range of microbiological enzymatic functions in relation to the resilience indicators.

In both indicators, LnVar and MaxArea, the most significant enzymatic activities were associated with the malonate cycle. Specifically, *Malonate decarboxylase holo-[acyl-carrier protein] synthase* (EC:2.7.7.66 as well as Malonyl-S-ACP decarboxylase (EC:4.1.1.87) and *Acetyl-S-ACP:malonate* ACP transferase (EC:2.3.1.187)) were identified. These enzymatic activities involved in the malonate cycle contribute primarily to lipid metabolism.

*NADH peroxidase* (EC:1.11.1.1) exhibited higher abundance in less-resilient animals, with an LFC value of approximately 0.8 for MaxArea and 0.3 for LnVar. This enzymatic activity along with A*ryl-alcohol dehydrogenase* (EC:1.1.1.90) belonged to the class of oxide reductase.

*Sugar phosphatase* (EC:3.1.3.23) involved in various metabolic pathways showed higher abundance in less-resilient animals, followed by *UDP-N-acetylglucosamine kinase* (EC:2.7.1.176).

Enzymatic activities more abundant in more resilient animals included those involved in the pathways of *Maleate isomerase* (EC:5.2.1.1), as well as 1*-aminocyclopropane-1-carboxylate deaminase* (EC:3.5.99.7), both associated with cysteine and methionine metabolism. These enzymatic activities exhibited LFC values of approximately −0.4 for MaxArea and −0.25 for LnVar.

Finally, *Sulfoacetaldehyde dehydrogenase (acylating)* (EC:1.2.1.81) showed a positive LFC and was exclusively present in LnVar, whereas *Sulfopropanediol 3-dehydrogenase* (EC:1.1.1.308) was observed only when considering MaxArea as a resilience indicator.

In the subsequent phase of our investigation, we extended those analyses (i.e., α-diversity, PERMANOVA and DA) by categorizing the resilience measures into three distinct groups: highly resilient groups (H), lowly resilient groups (L), and a middle group (M) acting as the control. This categorization was performed to amplify the differences observed at the extremes of the distribution of resilience phenotypes. By binning the resilience measures into these classes, we aimed to enhance the resolution and capture more pronounced distinctions between animals with high and low resilience. We defined the H animals as those exhibiting phenotypic values below the 5th percentile of the remaining animals within their respective breeds. Conversely, the L animals were defined as those with values exceeding the 95th percentile threshold of their breed group. Animals falling within the range of 47.5 to 52.5% percentiles of each breed were classified as the M, which served as the control group in our analyses. Performing this procedure within breed allowed to balance the design by breeds, alleviating differences among them. Since the Room factor was not used for defining the allocation to the resilience classes, we acknowledge a potential pitfall in this selection. However, the breed effect resulted as being more impactful on microbial composition than room (Supplementary Material 2).

Consistent with our previous PERMANOVA analysis, we observed significant variations in microbiota composition among the different classes derived from all resilience indicators (see Supplementary Material 5). Interestingly, when comparing the M to either the L or H using PERMANOVA, we found that there were no significant differences when transitioning from the M to the H for all resilience indicators. However, when transitioning from the M to the L group, significant differences in microbiota composition were observed for Lag1, LnVar, and MaxArea (*P* < 0.001). This suggests that the changes in microbiota composition may be more pronounced in animals with lower resilience compared to the more resilient ones, and the relationship between resilience and microbial composition might be non-linear.

**Fig. 7:**
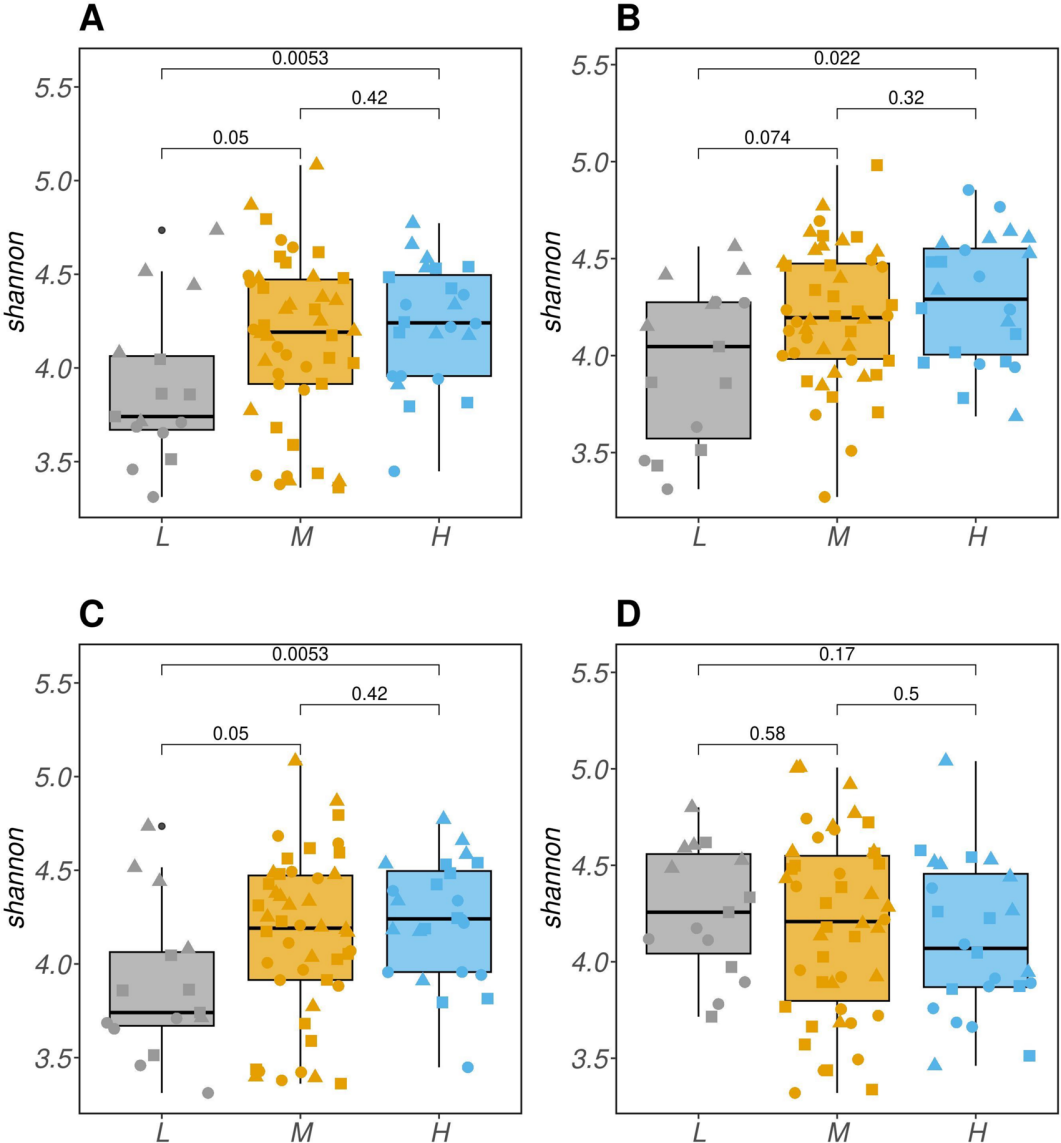
Shannon alpha diversity was assessed for the four resilience indicator were lag of 1 day of residual (**A**) natural logarithm of residual variance (**B**) area under the curve for periods with largest consecutive negative errors (**C**) and sum of residual’s local minima (**D**). The x-axis represents the resilience classes: lower (L), medium (M, as the control group), and higher (H). Above the plot, the *P*-values of the Kolmogorov-Smirnov test between the classes are reported above the boxplot. The shape of the point represents different breed within each class

The analysis of α-diversity further confirms the observed trends. Figure 7 shows that significant differences in α-diversity (*P* < 0.001) were observed among resilience classes. Notably, these differences were observed solely when transitioning from M or H classes to the L class. To simplify the presentation, only the Shannon index for α-diversity was reported in Figure 7, while the results for the inverse Simpson index can be found in Supplementary Material 6.

These findings align with the results obtained when resilience was considered as a continuous covariate, indicating that lower α-diversity is associated with lower resilience in animals.

**Fig. 8:**
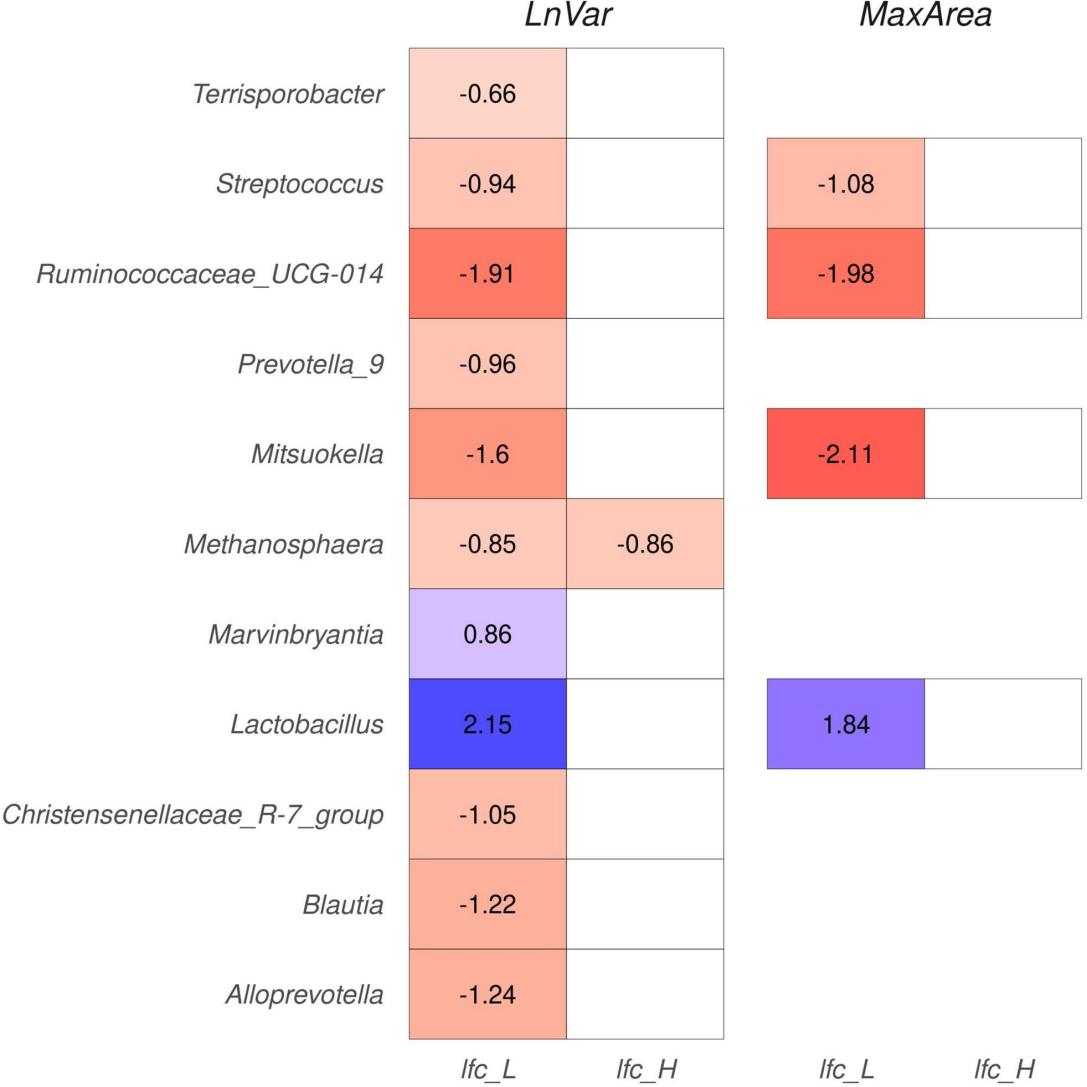
Heatmap illustrating the absolute value of the log fold change (LFC) abundances for the significantly abundant amplicon sequence variants (ASVs), with the indicators of natural logarithm of residual variance (LnVar) and area under the curve for periods with consecutive negative errors (MaxArea). The ASVs are grouped based on the genera to which they belong. The x-axis represents the LFC when comparing the resilient animal class with control groups (lfc_L), and the higher resilient animals class with control groups (lfc_H)

The DA analysis was also performed using class-based expression of different resilience indicators instead of linear variables. The results overlapped with the previous findings, both for individual ASVs and KEGG pathways, see Supplementary Material 7. Interestingly, the observed differential abundance of ASVs was primarily driven by comparisons between the less-resilient animals and the control groups, rather than comparisons between the more resilient animals and the control group. For example, in Figure 8, it is possible to observe that that no significant ASVs were identified when comparing the H and M groups over MaxArea.

We conducted a similar analysis to explore differences in the abundance of amplicon sequence variants among various classes of the four resilience indicators. The results were consistent with the previous analysis when resilience was considered as a linear variable. These analyses provided additional insights: the observed differential abundance of ASVs was primarily driven by comparisons between the less-resilient animals and the control groups, rather than comparisons between the more resilient animals and the control group. In fact, no significant ASVs were identified when comparing the H and M groups.

After identifying the connection and understanding the biological basis between gut microbiome composition and resilience, our next objective was to quantify the extent to which the microbiome contributes to the regulation of resilience. We aimed to determine the proportion of phenotypic variance in resilience that can be attributed to the microbial composition, which is commonly referred to as “microbiability” (m^2^) [29].

To address the influence of both environmental conditions and genetic background on resilience, we incorporated them into our analysis. Environmental conditions were accounted for by including the combination of the room and pen as a factor in the model. Additionally, the genetic background was considered by incorporating a sire effect in the model. While our primary focus was to quantify the impact of microbiota composition on resilience, we also provide a brief overview of the influence of these other factors on resilience.

**Fig. 9:**
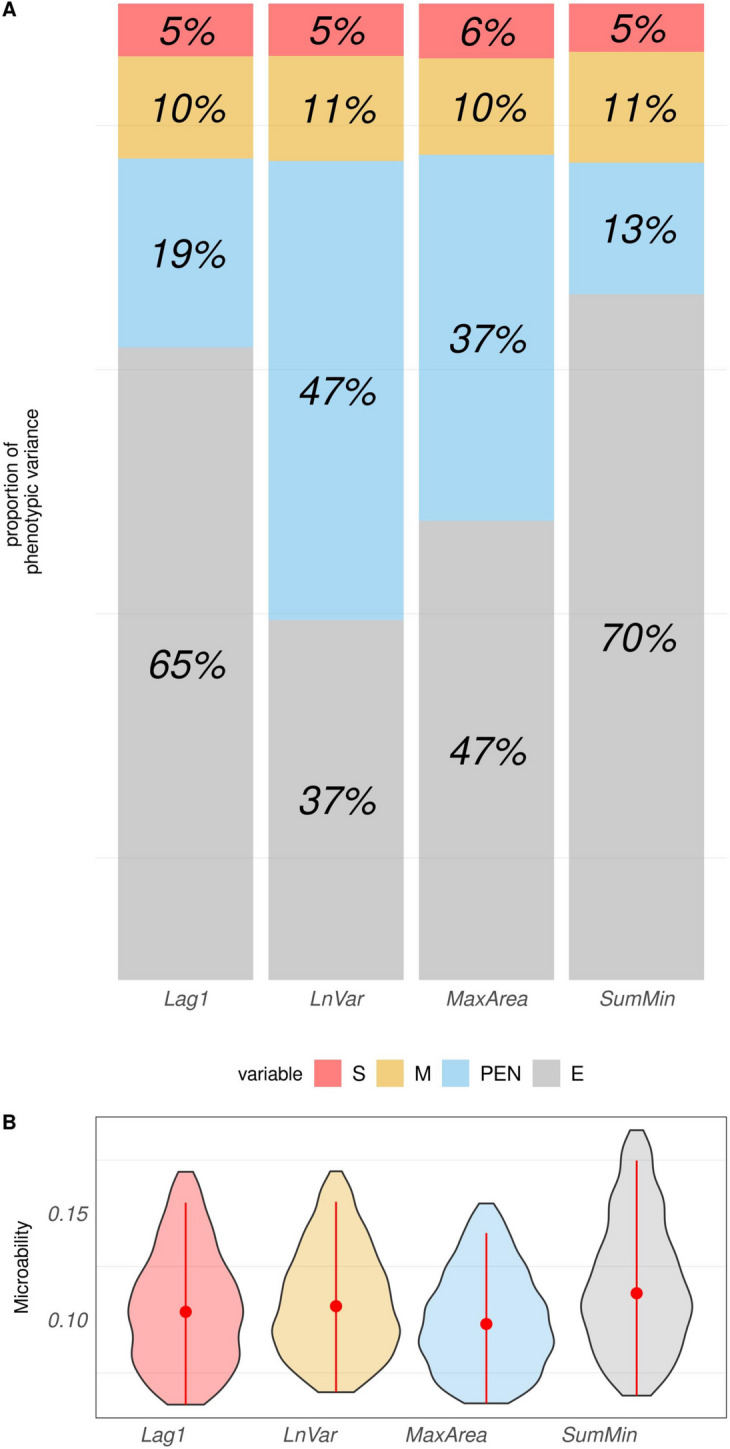
**A** Barplot representing the proportion of the phenotypic variance of the four phenotypes explained by the nested effect of sire (S) in red, microbiome (M) in yellow, pen (PEN) in light blue, and the residual error in grey (E). The four indicators are lag of one day of residual (Lag1), natural logarithm of residual variance (LnVar), area under the curve for periods with the largest consecutive negative errors (MaxArea), and sum of residual’s local minima (SumMin). **B** Violin plot of the posterior density distributions for the microbiability parameter (as the proportion of variance explained by the microbial effect), corresponding to the four resilience indicators mentioned above. The red dot represents the median of each posterior density distribution. The red line represents the HPD_95_ interval, which provides a measure of the uncertainty associated with the microbiability estimate

The percentage contribution of each factor to the overall variance in the analysis is depicted in Figure 9. Microbiota compositions explained a similar amount of variance for all resilience indicators, ranging from 10% for Lag1 and MaxArea to 11% for LnVar and SumMin. The highest posterior density intervals (HPD_95_) of m^2^ were reported in Part B of Figure 9. For the first three indicators, the HPD_95_ intervals ranged from 0.04 to 0.16, while a wider HPD_95_ interval of 0.04 to 0.19 was observed for SumMin. Across all resilience indicators, the genetic background of the animals accounted for approximately 5–6% of the phenotypic variance. However, the influence of the environment varied depending on the specific indicator. For Lag1 and SumMin, the Pen explained approximately 14 to 20% of the total phenotypic variance. In the case of MaxArea and LnVar, the environment contributed significantly more, accounting for approximately half of the total phenotypic variance. Specifically, the environment explained 37% of the phenotypic variance for MaxArea and 47% for LnVar.

Finally, we evaluated the discriminative capability of the microbiological data across the different classes of resilience indicators, we conducted a Partial Least Squares-Discriminant Analysis (PLS-DA). This analysis is of practical significance as it allows us to utilize microbiome composition as a potential biomarker for distinguishing and predicting animals based on their resilience levels.

Overall, our findings align with previous observations. The PLS-DA analysis demonstrated significant *P*-values (*P* < 0.05) for all resilience indicators, except SumMin (*P=*0.293), when assessing the ability to discriminate less-resilient animals. However, there were no significant differences in the discriminative ability when comparing the medium or high resilience classes.

**Fig. 10:**
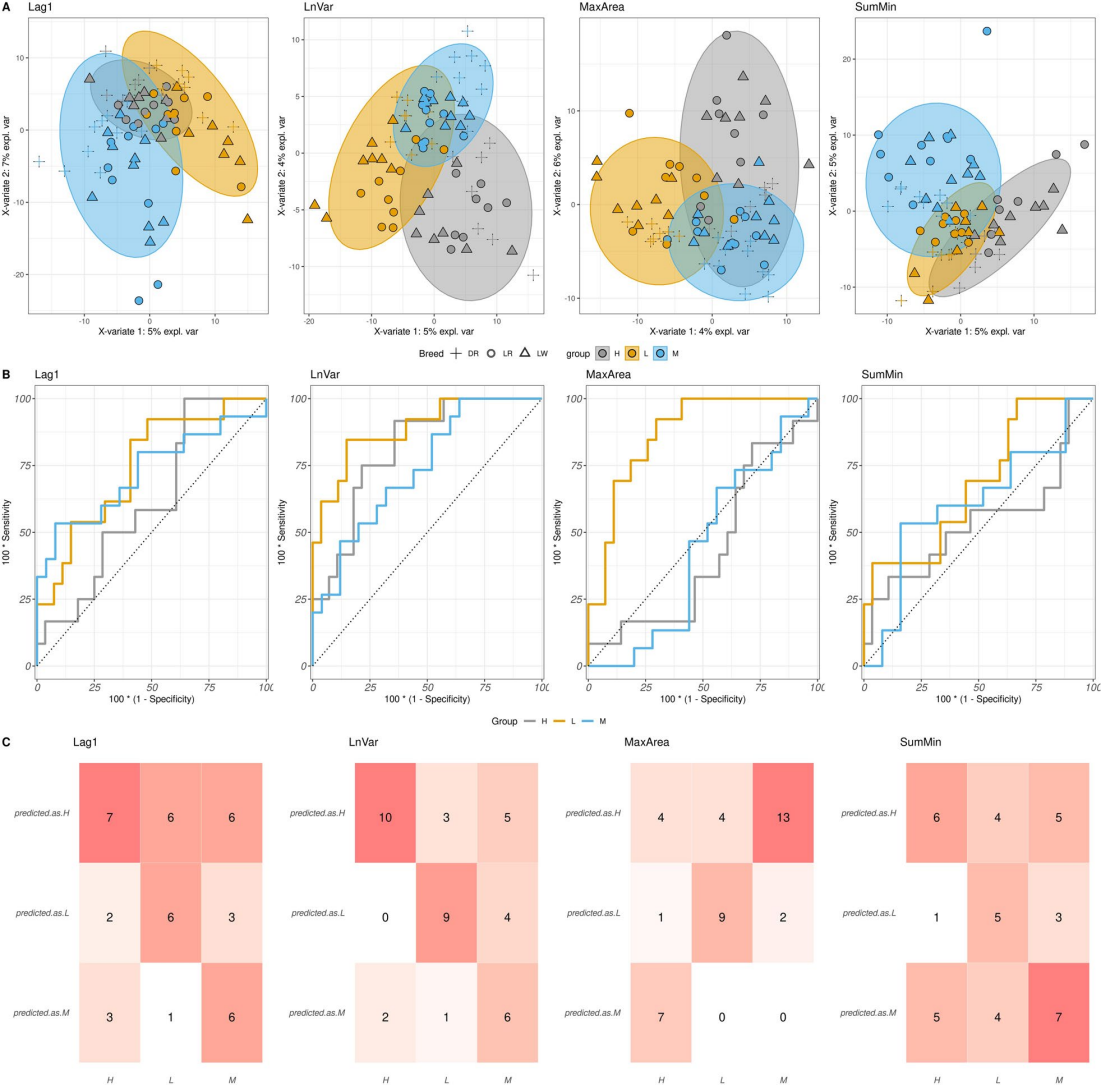
**A** A two-dimensional Partial Least Square-Discriminant Analysis (PLS-DA) score plot was constructed using three classes of resilience (lower (L) in yellow, medium (M) in light blue as the control group, and higher (H) in grey). The plot represents the distribution of the samples based on the first two components in the model. Each point’s shape changes according to the breed to which the animal belongs. PLS-DA was performed for the four indicators that are lag of 1 day of residual (Lag1), natural logarithm of residual variance (LnVar), area under the curve for periods with the largest consecutive negative errors (MaxArea), and sum of residual’s local minima (SumMin). **B** Receiver operating characteristic (ROC) analysis was performed to discriminate between the three classes of resilience mentioned above (L,M,H) for the four indicator of resilience mentioned above (Lag1, LnVar, MaxArea, and SumMin). **C** Confusion matrix depicting the performance of Lag1, LnVar, MaxArea, and SumMin in predicting the resilience classes (L,M,H). The x-axis represents the categories of true values, while the y-axis represents the categories of predicted values

The results of the analysis, illustrated in Figure 10A, revealed distinct clustering patterns for the H and L resilience classes in relation to the LnVar and MaxArea indicators. This suggests a clear differentiation between animals with high and low resilience levels. However, for SumMin, the separation between the classes was less pronounced, and no clear differentiation was observed for SumMin. Furthermore, the control class M did not exhibit complete separation and appeared to be positioned between the high and low classes in terms of LnVar and MaxArea. A stronger, but still not significant, separation was observed between the control class and the high and low classes in the case of Lag1.

Additionally, individuals of different breeds were equally distributed among the different resilience classes, indicating the absence of breed-specific patterns of resilience.

The Receiver Operating Characteristic (ROC) plot (Fig. 10B) reinforces our earlier observations, underscoring the heightened efficacy of microbiome composition in discerning distinct resilience classes. This effect was particularly prominent, especially for MaxArea and SumMin. This trend was further accentuated when Partial Least Squares-Discriminant Analysis (PLS-DA) was performed within specific breeds (refer to Supplementary Material 8). Moreover, the confusion matrix further supported (Fig. 10C) these findings, with a higher number of correctly identified instances in the low resilience group across all indicators. The values presented in Table 2 support the previous statement. With the exception of SumMin, the other resilience indicators demonstrated significant ability to discriminate low resilience animals. This was particularly evident for LnVar and MaxArea, which had *P*-values of <0.001. Lag1 showed a *P*-value of 0.01, along with AUC values of 0.726, 0.826, and 0.891 for Lag1, LnVar, and MaxArea, respectively. However, the AUC for discriminating the low-class using SumMin was 0.555.

**Table 2:** Performance of the Partial Least Squares-Discriminant Analysis (PLS-DA). The table provides the classification results for the four resilience phenotypes: lag of 1 day of residual (Lag1), natural logarithm of residual variance (LnVar), area under the curve for periods with the largest consecutive negative errors (MaxArea), and sum of residual’s local minima (SumMin). The “Class” column represents the classification of animal groups within each indicator, indicating low resilience (L), high resilience (H), and medium resilience (M). The “AUC Class” column displays the area under the curve (AUC) values for distinguishing each class of resilience. “Incidence” shows the proportion of records in corresponding resilience class. For each class, the table reports the number of positive events (animals belonging to that class) and negative events (animals not belonging to that class). “*P*-values” column indicates the significance level of the classification. The “Overall AUC” column represents the AUC performance in discriminating all classes combined

**Indicator**	**Class**	**AUC class**	**Incidence**	***P*-values**	**AUC all classes**
**Lag1**	L	0.726	0.325	0.010	0.693
	M	0.682	0.375	0.028	
	H	0.672	0.300	0.044	
**LnVar**	L	0.826	0.325	>0.001	0.726
	M	0.629	0.375	0.091	
	H	0.729	0.300	0.011	
**MaxArea**	L	0.891	0.325	>0.001	0.691
	M	0.637	0.375	0.078	
	H	0.544	0.300	0.336	
**SumMin**	L	0.555	0.325	0.293	0.617
	M	0.658	0.375	0.050	
	H	0.639	0.300	0.086	

Interestingly, despite the *P*-values for classifying the M and H classes being close to significance at 0.05 and 0.08, respectively, SumMin was the only indicator in which the microbiome exhibited superior ability to discriminate between the M and H classes compared to the L class. This finding suggests a negative correlation between SumMin and the other indicators. Consequently, higher values of H resilience could potentially indicate the contrary, i.e., lower overall resilience in this context.

Lag1 was the only indicator in which microbiome could significantly discriminate the M and H classes, while in LnVar microbiome significantly discriminated the H class from the others with a *P*-value of 0.011. When considering the AUC for discriminating all categories, LnVar had the highest value of 0.726, followed by Lag1 and MaxArea with values of 0.693 and 0.691, respectively. Lag1 had the lowest AUC value of 0.617.

In summary, we can conclude that all indicators of microbiome composition were effective in discriminating low resilience animals from the rest of the population, except for SumMin, for which higher values represent higher animal resilience. Among these indicators, MaxArea supported the best performance, while LnVar exhibited the best overall classification performance in terms of microbiome composition.

## Discussion

Our study yielded four notable findings. Firstly, we successfully identified reliable indicators of resilience by analyzing variation in daily feed intake in pigs. Secondly, we discovered a strong relationship between microbial composition and resilience. Moreover, we identified specific ASVs and KEGG pathways that are in association with inflammatory response and linked to resilience. Thirdly, our results suggest that differences in microbiome composition are primarily observed in animals with lower resilience. Lastly, our study suggests that microbial composition has the potential to serve as a dependable biomarker for distinguishing individuals with lower resilience.

In our investigation, we focused on the relationship between feed consumption variability and animals’ resilience. Our findings align with previous studies conducted in mice, which have shown that abnormal feed consumption, particularly reductions, can be attributed to various negative and traumatic events [16]. These events encompass psychological states influenced by environmental factors [30] or genetic factors [31], as well as physiological conditions such as tumors [32] and microbial infections [33]. Therefore, fluctuations in feed consumption can serve as indicators of these underlying factors.

In this study, we captured these fluctuations accurately, by continuously monitoring and recording of feed consumption. This allowed prompt identification of variations thus improving the precision of event identification and assessment of animals’ response capabilities [34]. However, not all indicators examined in this study proved effective in capturing the fluctuation or variability in feed consumption. Among the indicators considered, LnVar and MaxArea emerged as successful indicators. While LnVar has been previously acknowledged as a suitable indicator, particularly in the livestock sector [35–37], the suitability of MaxArea as an indicator had not been previously explored.

Notably, we observed a modest antagonistic correlation between the two indicators (LnVar and MaxArea) and the percentage of muscle. This observation strengthens our hypothesis that animals displaying higher variability in feeding behavior tend to have lower resilience. The percentage of muscle was utilized as a surrogate measure for a 'wellness' trait, as animals in better health generally exhibit a higher muscle mass relative to their body weight [38]. This finding provides additional support for the notion that animals with greater fluctuation in feed consumption may have compromised overall health and resilience.

Previous research has emphasized the difficulties in identifying a specific indicator of animal resilience in the absence of deliberate stress-inducing experiments, such as natural disease challenges [13, 39]. However, a noteworthy study by [40], which focused on divergent selection for variability in rabbit litter size, challenges this notion. Subsequent investigations on this particular population, employing genomic analysis [19] and metabolic profiling [21], unveiled a link between this variability and a more comprehensive notion of animal resilience. These findings align with later studies that also observed associations between genomic profiles and resilience, even in the absence of intentional stress induction, as in our current study [35, 41].

The primary objective of this study was to explore the correlation between gut microbial composition and the resilience phenotype. Prior research has already established the link between gut microbiome composition and diverse phenotypic expressions [42], including traits related to livestock production [43]. The microbiome acts as an intermediary layer between genetics [44] and environmental factors [45]. This intricate relationship, coupled with the dynamic response of the microbiome to external stimuli [46], positions it as a valuable tool for enhancing our understanding of complex traits, such as resilience [42].

Consequently, recent investigations have prioritized unraveling the association between the microbiome and resilience. Studies have examined resilience in various contexts, encompassing the resistance of coral to climate changes [47, 48], the practical implications of microbiome engineering in plants [21], and the role of microbial composition in thermal stress adaptations across different animal species, including tadpoles [49], *Drosophila* [50], and mice [51]. However, within the domain of livestock, only one study conducted by Casto-Rebollo et al. in 2023 in rabbits has explored the relationship between the microbiome and resilience [22]. In contrast with such study [28], which investigated changes in microbiome composition resulting from shifted selection for resilience and viewed the microbiome as an intermediary layer between host genetics and phenotypic expression, our study took a distinct approach. Our objective was to elucidate the relationship between microbiome composition and resilience, considering both genetic and environmental factors as driving forces for these variations. Despite these differences, there are notable similarities that have emerged between the studies. Both studies have highlighted the capacity of microbial composition to differentiate between resilient and less-resilient animals, as well as the involvement of KEGG pathways associated with the inflammatory system in the context of resilience. However, our study also revealed significant discrepancies in terms of α-diversity and β-diversity measures, which were not observed in the aforementioned studies. These disparities could be attributed to several factors, including variations in sample size, the specific focus of the studies, and the narrow time window between microbiome collection and resilience assessment employed in our investigation. These factors likely contributed to the divergent findings between our study and the previous research.

In terms of α-diversity, our findings revealed a notable decrease in microbial richness among animals with lower resilience, particularly within the subset of less-resilient individuals. This decline suggests two potential interpretations. Firstly, microbial richness within the composition may function as a reservoir to counteract and respond to external events [52]. Secondly, the reduction in α-diversity could be indicative of an unhealthy state in the animals, such as bacterial infection [53], reflecting their limited restorative capacity.

Moreover, our study identified a strong and significant association between β-diversity, assessed using PERMANOVA, and resilience. This outcome underscores the clear connection between microbial composition and resilience, providing further evidence for the relationship between microbial compositions and complex traits such as resilience.

Moreover, intriguing findings were obtained through different differential abundance (DA) analyses. Particularly, the ANCOM-BC analysis identified amplicon sequence variants (ASVs) with significantly distinct abundances. Of particular interest was the observation of higher abundances of ASVs affiliated with the *Lactobacillus* genus in animals exhibiting lower resilience. At first glance, this finding may seem contradictory, given the well-documented beneficial effects of various *Lactobacillus* strains on swine, including enhanced productivity [54, 55] and improved immune function [56].

To explain this observation, several hypotheses can be considered. One possibility is that the identified ASVs represent specific strains of *Lactobacillus* that possess potential pathogenic properties [57]. However, it is important to note that this hypothesis remains speculative and requires further investigation. Another plausible explanation is that *Lactobacillus*, being an enterotype bacterium in swine [58, 59], may encounter reduced competition from other bacterial species in animals with lower resilience, leading to its higher abundance.

Alternatively, the increased abundance of *Lactobacillu*s in animals with lower resilience may be associated with the promotion of cytokine production [60], resulting in an inflammatory response [61] aimed at preventing and combating infections. Therefore, the higher abundance of *Lactobacillus* in such animals could potentially serve as a biomarker for ongoing inflammatory processes [62], indicating the organism’s efforts to counteract negative events.

Among the genera that exhibited greater abundance in more resilient animals, *Ruminococcaceae-UCG-014* stood out as the most prominent. Extensive studies on mice have consistently demonstrated the beneficial effects of *Ruminococcaceae-UCG-014* on gut health. Bacteria belonging to this genus have been shown to significantly increase the production of short-chain fatty acids (SCFAs), such as acetate and propionate, which play critical roles in improving gut health, regulating cytokine levels, and modulating the gut microbial community [63].

Furthermore, it has been observed that supplementation with the amino acid Glycine (Gly) promotes the growth of both *Ruminococcaceae-UCG-014* and *Ruminococcaceae-UCG-03* (found more abundant among resilient animals in this study), leading to enhanced intestinal healing in mice [64]. In addition, studies on pig diets have also demonstrated that supplementing with Gly amino acids can increase the abundance of *Mitsuokella* and *Prevotella_9* (two other genera more abundant in more resilient animals), leading to increased production of SCFAs, including lactate, acetate, and propionate [65]. Additionally, our study identified *Oscillospira* as more abundant in resilient animals. This finding aligns with recent research suggesting that *Oscillospira* functions as a “next-generation probiotic” capable of producing SCFAs, particularly butyrate, which is known for its important role in promoting gut health [66].

The ANCOM-BC analysis performed on KEGG pathways supports our findings from the preceding section. We identified a notable increase in the prevalence of NADH peroxidase in animals exhibiting lower resilience, landing support to our initial hypothesis that the heightened abundance of *Lactobacillus* in less-resilient animals might be linked to an inflammatory status in the animal. Indeed, NADH peroxidase plays a critical role in eliminating potentially harmful hydrogen and reducing oxidative stress, which can arise as a result of disease conditions [67]. Therefore, the elevated abundance of NADH peroxidase, particularly in *Lactobacillus*, may indicate the organism’s endeavors to combat such conditions. This interpretation is supported by previous studies reporting the presence of NADH as an indicator of infection within 1–24 h [68].

Aryl-alcohol dehydrogenase, another pathway more abundant in less-resilient animals, belongs to the oxidoreductase family, similar to NADH peroxidase. Based on its involvement in a wide range of biological processes (https://www.brenda-enzymes.org), this suggests that aryl-alcohol dehydrogenase may also play a role in mitigating oxidative stress, similar to NADH peroxidase. However, further investigation is needed to fully understand the specific roles and implications of aryl-alcohol dehydrogenase in resilience.

Sugar phosphatase was also found to be more abundant in animals with lower resilience. This enzyme catalyzes the production of sugar from sugar phosphate. One possible explanation for its presence in less-resilient animals is its role as a source of energy for T-cells, i.e., important types of white blood cells of the immune system [51]. Normally, T-cells exhibit low metabolic activity and rely on fatty acid oxidation for energy. However, when T-cells are activated, they undergo rapid proliferation and differentiation into effector T-cells, requiring increased glucose metabolism and glycolysis [69]. Therefore, the higher abundance of sugar phosphatase in less-resilient animals may be a consequence of disease conditions, similar to what identifies for NADH and *Lactobacillus*.

Furthermore, we identified three out of seven main KEGG pathways involved in malonate metabolism that were more abundant in animals with lower resilience. Although clear evidence is lacking, experimental studies conducted on mice have demonstrated that malonate injections can impair the mouse’s defense against infection [70]. One hypothesis suggests that the utilization of malonate by *Pseudomonas aeruginosa* affects quorum sensing and virulence and promotes the formation of mineralized biofilm-like structures [71].

Among the KEGG pathways that were more abundant in resilient animals, we identified 1-aminocyclopropane-1-carboxylate deaminase as one of the most prominent pathways. Interestingly, while the beneficial role of this pathway in plant resilience has been well-established [72], no previous studies have identified its significance in animal resilience.

Another pathway that showed higher abundance in resilient animals was the maleate isomerase. Previous studies have suggested the potential benefits of maleate isomerase in overcoming challenges during the post-weaning period in pigs [73], as it involved in the metabolism of precursor acids such as citric acid, formic acid, fumaric acid, lactic acid, or propionic acid.

Our findings, partially in line with a previous study [74], initially reveal distinct patterns in swine deemed less “resistant” to social stress, showing diminished performance and reduced feed consumption. Additionally, the study highlights that resilient animals exhibit a higher abundance of genera linked to short-chain fatty acid (SCFA) production, such as *Prevotella* and *Mitsuokella*. However, differences emerged between our study and the aforementioned one regarding the genera identified in less-resilient animals. In our investigation, *Lactobacillus* was more prevalent, whereas others [74] observed elevated levels of *Clostridium* and *Campylobacter*. These variations may be attributed to differences in the experimental designs of the two studies.

In our study, the simultaneous measurement of resilience and microbiota revealed that less-resilient animals are actively combating infections or diseases, with *Lactobacillus* playing a crucial role. Conversely, in the earlier study, microbiota samples were collected post-stress, resulting in an increased abundance of specific genera. Nonetheless, our study did identify significant differences in alpha diversity between the stress and control groups.

In addition to investigating the relationship between the microbiome and resilience, our study aimed to quantify the contribution of microbial composition to resilience by calculating the proportion of overall indicator’s variance attributable to the microbiome. Recently, microbiability has emerged as a parameter that allows us to quantify the extent to which variation in host-measurable variables can be ascribed to the gut microbiota in agricultural animal species [29]. Assessing the percentage of resilience variation explained by the microbiome is crucial as no previous studies, to the best of our knowledge, have quantified the impact of the microbiome on complex features like resilience. Furthermore, if microbial composition demonstrates a significant impact on controlling resilience, it could potentially pave the way for utilizing the microbiome as a tool for improved prediction and selection of resilience [26, 28]. This is particularly relevant for complex traits like resilience, which are characterized by low heritability and controlled by multiple genes, posing challenges in terms of selection [75].

Our study provides evidence for the significant role of microbiome composition in explaining the variation observed in resilience. Specifically, we found that the microbiome accounts for more than 10% of the total variance in resilience. Although the magnitude of the microbiome’s influence (m^2^) on resilience may appear lower compared to studies investigating other traits such as feed efficiency, fat/muscle deposition, and feed behavior (which typically range from 10 to 30%) [25, 76, 77], it is important to consider the context of resilience as a trait with inherently low heritability (5–10%). Thus, the microbiome represents a substantial and meaningful source of variation for resilience. It should be kept in mind, though, that our analysis considered the microbial component independent from sire (genetic) and pen (environmental) effects, which was forced by the data structure. This should be addressed in further studies, since the components should not be considered as independent, both conceptually and practically.

Finally, when we employed the entire microbial composition, we were able to identify animals accurately with lower resilience. When considering the most reliable resilience indicators, LnVar and MaxArea, our results were consistent with a previous study conducted by Casto-Rebollo et al. [20]. While low resilience animals were identifiable leveraging their microbial composition, PLS-DA analysis was not able to distinguish more resilient animals from those with average resilience, aligning with our earlier findings that did not reveal significant differences in microbial compositions between these two groups. Interestingly, despite the known influence of breed on microbial compositions [78], we did not know if there were any breed-related interference in discriminating resilience in PLS-DA. Observing the results of the PLS-DA was possible to observe that all the breeds was balanced across the resilience classes, suggesting that the link between microbial composition and resilience is not strongly influenced by breed. These findings carry practical implications, as the microbiome can serve as a cost-effective tool for monitoring the health status of animals and their ability to respond to adverse events, thus reflecting their resilience [79].

## Material and methods

### Data

#### Animals and feed data

The data used for this study come from the performance test station owned by Smithfield Premium Genetics (Rose Hill, NC, USA). The station housed part of the nucleus herd and was maintained under biosecurity conditions, suitable for a breeding herd. The animals could not be deemed free from any infectious disease despite these measures. Data were generated between May and December 2017. Animal in the trial belonged to three swine breeds: DR boars (*N* = 190), LR boars (*N* = 221), and LW boars (*N* = 204). These animals were the offspring of 27, 27, and 44 sires, respectively, and were crossed with 119, 153, and 158 dams for the DR, LR, and LW breeds.

During the growth trial, the pigs were provided with standardized pelleted feed and received standard vaccinations and medications. The trial took place on a nucleus farm consisting of eight rooms, where the animals were housed in separate groups according to their breed. On average, each group contained 11.3 ± 1.3 animals, each group was allocated in a different physical pen and there were eight pens per room. Full-sibs and paternal half-sibs were allocated into different pens and rooms, so that the sire and pen effects were not confounded. This was ensured at every round of pen allocation, since this data was used for comparing sires’ offspring and making selection decisions. To monitor feed consumption, each group was equipped with a single-space Feed Intake Recording Equipment (FIRE) feeder from Osborne Industries, Inc. (Osborne, KS, USA). The FIRE feeder recorded the feed consumption of pigs for each visit to the feeder, capturing the pig’s identifier and its weight. Feed consumption was the raw measure used to calculate the resilience indicators in this study.

#### Microbial composition

Fecal samples were taken at the beginning, end and mid-time of the trial as described in [25, 80], DNA extraction was performed on rectal swabs using the method described in detail by Lu et al. [81]. For sequencing, the V4 region (515-806) of the 16S rRNA gene was amplified in a phased and bi-directional manner to generate indexed libraries suitable for Illumina sequencing, following the previously described protocol [81]. The sequencing process was carried out at the DNA Sequencing Innovation Lab, located at the Center for Genome Sciences and Systems Biology at Washington University in St. Louis (USA).

The raw sequence data generated by the Illumina platform were converted into read files using MiSeq Reporter. To merge pairs of V4 16S rRNA gene sequences, FLASH v1.2.11 [82] was employed, requiring a minimum overlap of 100 base pairs and a maximum of 250 base pairs for confident overlap. The resulting sequences were oriented in the forward direction, and any primer sequences were trimmed, allowing for up to 1 mismatch during primer matching.

The sequences were imported into Quantitative Insights Into Microbial Ecology (QIIME2 version 2017.12, https://qiime2.org/) for demultiplexing. The construction of an amplicon sequence variant (ASV) feature table was performed using the Divisive Amplicon Denoising Algorithm 2 (DADA2) [83] with default settings, without truncation or length filtering (--p-trunc-len 0). ASVs present in only one sample were removed from the feature table.

Furthermore, taxonomic information was annotated using the Ribosomal Data- base Project (RDP) Classifier (v2.4) based on the SILVA reference database (v132) [84] and applied to predict taxonomic assignments for each ASV sequence, using a confidence cutoff of 0.8. The results were exported for further analysis in the R environment [85]. Second-level and third-level ontology pathways of the Kyoto Encyclopedia of Genes and Genomes [86] predictions were obtained using the PICRUSt2. Software [87].

### Variability as resilience indicator

#### From single visit to daily data

To estimate different indicators of animal resilience, we utilized daily feed consumption (FCD) as the phenotype. FCD was determined by summing the feed consumption for each visit occurred over a 24-h period. Prior to converting the individual visit data into daily data, we applied a data cleaning procedure based on the method developed by Casey et al. [88], which was adapted to suit our specific data requirements reported on Supplementary Material 9.

We analyzed the data collected during the time period of 99 to 140 days, which was referred to as the “mid-period” in the study by He et al. [25]. Briefly, feeding records were categorized into three periods (P1: 73 ± 3 to 98 days of age; P2: 99 to 140 days of age; P3: 141 to 163 ± 6 days of age), corresponding to the three time points of rectal swab collection (T1: 73 ± 3 days of age; T2: 123 ± 4 days of age; T3: 158 ± 4 days of age). The breakpoints at 98 and 140 days of age demarcate the midpoints between T1 and T2 and between T2 and T3 of rectal swab collection.

The microbiota samples were specifically collected at an average age of 123 ± 4 days. Focusing on this specific time window allowed us to mitigate any potential influence of the time elapsed between the collection of the phenotype data and the microbiota data, ensuring a more accurate assessment of the association between the two.

After incorporating the FCD data, we conducted a subsequent round of data editing. This iterative editing process involved removing animals with fewer than 10 FCD observations and those with more than 3 consecutive days of missing FCD data. The final dataset comprised 528 animals (DR: *N*=153, LR: *N*=193, LW: *N*=182) with an average of 41.4±1.2, daily record per each animal. For a comprehensive understanding of the data-editing procedure, please refer to Supplementary Material 9 for detailed information.

#### From daily data to resilience indicator

The resilience indicators were determined based on the variability of their daily feed consumption (FCD) information. The rationale for this approach is that animals with greater variability in their daily feed intake may exhibit challenges in maintaining a stable physiological status [89].

To ensure the accuracy and reliability of our analysis, we took into consideration the potential influence of random daily fluctuations, commonly referred to as “white noise” (i.e., random fluctuation). In order to mitigate this effect, we applied a moving median approach with a window size of 5 days. This method was implemented using a custom R script.

Furthermore, we recognized that the larger variance in FCD could potentially be influenced by the daily increase in feed consumption due to the animals’ growth. To address this concern, we calculated the resilience indicator based on within-animal residuals. This involved subtracting the moving median FCD values from the values predicted using linear regression of FCD on age. A positive residual indicated that an animal’s feed consumption was higher than the expected consumption for that period, while a negative residual indicated the opposite. This approach allowed us to account for the influence of growth-related changes in feed consumption and focus on deviations from the expected trend.

From the residuals, we derived various indicators of animal resilience. However, it is important to note that there is no universally accepted indicator of animal resilience [90]. Therefore, we defined four different resilience indicators, each capturing a distinct aspect of “variability.”

One of these indicators is the autocorrelation with lag of 1 day (Lag1), which describes the level of dependence between an individual’s daily feed consumption data. We hypothesize that higher Lag1 values indicate greater independence between daily feed consumption data, which is therefore associated with increased variability [91].

Another indicator is based on the classical estimation of variance, calculated by calculating the variance of individual moving median FCD data (adjusted by age, as explained above). Since variance estimates are not normally distributed, we normalize it using the natural logarithm. That indicator was defined as LnVar [91].

The third indicator is based on the concept that a significant portion of the variance in feed consumption can be attributed to negative periods, which indicate a decline in feed consumption. These negative periods can be influenced by external factors such as illness or disease. To define this phenotype, we calculated the area under the curve for periods with consecutive negative errors using the definite integral. Among all individuals, we consider the area with the largest value as the third phenotype, which we refer to as MaxArea. As for LnVar, we transformed it using the natural logarithm.

Finally, the fourth indicator is based on the notion that “variability” arises from continuous fluctuations in feed consumption following a similar approach of Putz et al. [13]. This is quantified by calculating the sum of local minima, where each local minimum represents a point in the feed consumption data that is lower than all of its neighboring points. We refer to this indicator as SumMin.

### Association between microbial composition and resilience

The primary objective was to investigate the relationship between microbial composition and resilience. We approached this investigation by considering resilience as both a linear or categorical indicator. To analyze this relationship, we employed various analytical techniques, including PERMANOVA, α-diversity analysis, and different abundance analysis (DA).

#### Resilience as linear indicator

Permutational multivariate analysis of variance (PERMANOVA) was employed to investigate whether variation in microbiota composition was associated with changes in resilience indicator.

The PERMANOVA analysis was performed by considering microbiota composition as the dependent variable and each resilience indicator as a (covariate) independent variable. For each analysis, each resilience indicator was considered separately. Additionally, in each analysis the effects of room (with 8 levels) and breed (with 3 levels) were included in the analysis to account for their potential influence. While for what regarding microbial composition, we filtered the animals that has total count lower than 700, resulting a total of 502 animals (DR: *N*=145; LR: *N* =187; LW: *N* =170). Additionally, ASVs with a prevalence rate less than 0.05 were removed, resulting a total of 1.074 ASVs. Then ASV was transformed by the centered log-ratio transformation using the Tjazi package [92], where missing zero values were imputed using the *"const"* approach defined in [93]. That transformed ASV data was then used to compute the Euclidean distance using the *"dist"* R base function [85], representing the dissimilarity between samples and that was used as a dependent variable in the PERMANOVA analysis. The PERMANOVA analysis was conducted using the *"adonis"* function in the vegan package [94], with 1000 permutations.

Additionally, to examine the relationship between resilience and diversity in gut microbiota composition, we computed α-diversity for all animals (*n*=528) and all ASV (*n*=4595) of the microbiota without applying any filters. The α-diversity was calculated using inverse Simpson’s index and Shannon’s index calculated by using the “microbiome” R packages [95]. Observations deviating beyond 1.5 times the interquartile range above third quantile or below first quantile were classified as outliers and removed for subsequent analysis. To assess the significant influence of microbial composition diversity on resilience, *P*-values were calculated for the slopes of regressions between each resilience indicator and two α-diversity measures. Considering α-diversity as the dependent variable, each distinct resilience trait was treated as an independent variable. To further validate the influence of α-diversity on resilience, a linear regression was conducted, additionally accounting for the effects of room and breed, as outlined in Supplementary Figure 10. This analysis was performed using the *"lm"* function in R [85].

Furthermore, we were also interested to identify specific ASVs that exhibited significant differences in abundance between animals with different levels of resilience. Similarly to the previous analysis, we included only animals with a minimum total count of 700 (*n*=502), but no filter was applied to individual ASVs. The Analysis of Compositions of Microbiotas with Bias Correction (ANCOM-BC) [96] was utilized for this purpose, as microbiota data is compositionally natured and can be influenced by sampling and sequencing depth. ANCOM-BC addresses this challenge by accurately estimating and eliminating the bias introduced by differences in sampling fractions in the observed counts. The methodology utilizes relative abundances to infer absolute abundances while controlling the false discovery rate and addressing excess as zero counts. Similarly, for the PERMANOVA analysis, we considered each of the four phenotypes as a linear covariate, and breed and room as cross-classified effects. Due to the granularity of the microbiota compositions, no agglomeration was performed, and DA was considered at the ASV level. ASVs that exceeded the threshold of 0.05 *P*-values corrected by Bonferroni test were considered significant.

To examine the DA of various enzymatic activities (or KEGG pathways), we also employed ANCOM-BC. In this analysis, we utilized the KEGG-enriched pathways obtained as described in the “Bioinformatic analysis” section instead of single ASV. A total of 2026 KEGG pathways were identified. The analysis was conducted using the same criteria employed before. Each enzyme activity was treated as an independent variable, and we only included animals that met the cleaning parameter from the previous analysis, specifically animals with a total count of microbiota higher than 700.

#### Resilience as categorical indicator

As mentioned earlier, we also transformed the four resilience indicators into categorical variables. This modification allowed us to assess the non-linearity of the relationship between microbiota and resilience.

The definition of resilience classes in our study followed a specific procedure. Resilient animals were identified as those whose values for the target phenotype and breed were below the 5th percentile, indicating a higher level of resilience. Conversely, non-resilient animals were characterized by values exceeding the 95th percentile, indicating a lower level of resilience. Control groups were comprised of animals whose resilience indicators fell within the 47.5th and 52.5th percentiles, representing an intermediate level of resilience.

This classification was performed using the "*ntile*" function from the dplyr package [97]. Approximately 30–40 animals per breed were included, and the distribution of each resilience class within each group is presented in the Supplementary Material 11.

PERMANOVA analysis was performed followed a similar procedure as before, but this time we focused on testing the significance of these measures in relation to the resilience class, rather than as a linear indicator. In this analysis, PERMANOVA was conducted by considering all classes of resilience, as well as comparing the medium class with high resilience indicators and the low resilience class with the medium class (control group). In addition, α-diversity analysis was conducted using the same criteria as mentioned before. Here we assess different microbial richness of class by using the *P*-values which were computed using the Kolmogorov-Smirnov test [98].

DA was conducted using the same procedure as when resilience was considered as linear indicator. However, in this case, the LFC was computed based on difference between resilience classes, with the medium resilience group serving as the control class. Specifically, we calculated the LFC of low resilience animals compared to the medium resilience group, as well as the LFC of high resilience animals compared to the medium resilience group. Similar to the previous analysis conducted when resilience was treated as a linear indicator, the analysis was performed for both at ASVs and KEGG pathways levels.

### Microbiability estimation

To assess the influence of the microbiota on four different resilience indicators, we employed Bayesian Kernel linear regression. This approach allowed us to quantify the impact of the microbiota on resilience indicators by calculating the ratio between the estimated variance attributed to the microbiota and the total variance. This ratio, known as microbiability (m^2^), provides a measure of the microbiota’s contribution to the variation of a target indicator variable. As mentioned before, that analysis was conducted when resilience was considered as linear indicators.

In our analysis, we considered the effect of the microbiota by considering the similarity between animals based on their microbiota composition using an inner kernel. This approach is mathematically equivalent to multiple random regression (also known as ridge regression) but is computationally more efficient when the number of variables is larger than the number of samples [99], as in our case.

Before constructing the inner kernel, similar to the approach used in the PERMANOVA analysis, we applied certain criteria. We only considered animals with a total count higher than 700 and ASVs with a prevalence rate greater than 0.05, resulting in a dataset of 502 animals and 1074 ASVs. The ASVs were then transformed using the additive centered log-ratio transformation. Finally, the inner kernel was computed as the inner product of the scaled ASV matrix.

In accounting for the influence of microbiota (M) on resilience, we considered resilience as affected by environmental factors (E), signified by the pen (with 61 levels), the genetic background of the animals (S), represented by the animal’s sire (84 levels) nested within the breed (3 levels), and by random residual environmental effects. The sire by pen interaction was not estimated due to the design allocating full-sibs and paternal half-sibs to different pens. Additionally, we treated M as independent of the environmental and genetic background of the animals. Although we recognize this as an approximation [100], this approach enables a quantitative assessment of the proportion of variance captured by the microbiome that is independent of environmental conditions and genetic influences.

To model these effects, we assumed that M, E (environmental effects), S (genetic factors), and residual were normally distributed with a mean equal to 0 and a variance ${\sigma }_{M}^{2},$ ${\sigma }_{E}^{2}$, ${\sigma }_{S}^{2}$ ${\sigma }_{e}^{2}$ respectability. The variance of M was equal to the microbiota similarity matrix multiplied by the variance of the microbiota. We also assumed that the residuals were independently and normally distributed and that the M S, E, and residual effects were uncorrelated. The analysis was conducted using the BGLR R package [101].

The analysis involved running 70,000 iterations with a burn-in period of 10,000, and to reduce autocorrelations, we considered only 1 out of every 50 samples. We tested the convergence of the model using Geweke’s *Z* criterion [102]. The magnitude of the microbiota’s influence on resilience was calculated as the median of the posterior density distribution of m^2^, defined as (${\sigma }_{M}^{2}$/*sum*(${\sigma }_{M}^{2},$ ${\sigma }_{E}^{2}$, ${\sigma }_{S}^{2}$ ${\sigma }_{e}^{2}$)), and the highest posterior density interval at 95% probability (HPD_95_).

### Microbial composition to discriminate between resilient and non-resilient individuals

To determine the classification performance of the microbiota for different classes of resilience, we conducted PLS-DA. In this analysis, the three classes of resilient animals (resilient, non-resilient, and control) for each phenotype were treated as the target variable, while the centered log-transformed ASV data (with a frequency rate > 0.05) served as the feature data (*n*=1074). The data was randomly split into training and test by allocating 50% of data to each set by maintain equal allocation of data for each breed and resilience class.

PLS-DA was performed using the caret R package [103].

We trained a PLS-DA model, in two steps. The first step consisted in determining the optimal number of components through repeated 4-fold cross-validation with 50 repeats, with the number of components being chosen based on the AUC values obtained. After determining the optimal number of components, we performed feature selection by eliminating variables with lower contributions. Specifically, we removed the features that accounted for the lowest 20% of the variable importance, as measured by the variable importance in projection metric. The iterative process continued until the highest AUC value was achieved, indicating the best classification performance of the model. To evaluate the discriminant ability of the microbial composition, we used metrics such as AUC, ROC (receiver operating characteristic), and examined the confusion matrix on test populations. AUC was calculated both for the ability of the PLS-DA model to discriminate each element of the three classes and for the overall PLS-DA performance. For calculating the AUC in a multi-classification framework, we utilized the "multi.ROC" package [104] and the "MLmetrics" package [105]. The significance of the discrimination of individual classes was assessed using Wilcoxon tests with *P*-values below 0.05, employing the "verification" package and the "wilcox.test" function [106].

## Conclusion

The current study highlights a sizable link between the gut microbiota and resilience in swine. We developed an effective procedure to capture and estimate animals’ resilience, and linked it to the gut microbial composition. Based on our results, among all indicators, LnVar and MaxArea were the most effective in capturing the link with the microbial composition.

We observed variation in microbiome composition and diversity across different levels of resilience. Specifically, animals with lower resilience exhibited reduced richness in terms of α-diversity. Additionally, we identified specific ASVs and KEGG pathways associated with inflammatory responses, linked to the host’s efforts to mitigate negative events in these animals. Remarkably, these changes in the microbiome were primarily observed in animals with lower resilience.

With this work we had shown that microbial composition has the potential to serve as a reliable biomarker for distinguishing individuals with lower resilience, providing a cost-effective indicator of animals’ biological status. Moreover, microbiability analysis confirmed the active role of the microbiome in controlling resilience, offering a means to identify robust animals for achieving improved outcomes but also to identify animals more prone to suffer from stress, suggesting it as a possible way to improve animal welfare. As a result, these findings provide a foundation for leveraging valuable insights and tools from host-microbiota interactions to manage and enhance animal well-being and resilience.

### Supplementary Information

**Additional file 1:** **Supplementary Material 1. **Tuckey Test of Breed effect resulting from the mixed models analysis for the four different resilience indicators. **Supplementary Material 2.** PERMANOVA analysis for the effect each of the systematic effect of the Breed and Room on the host microbiota composition. **Supplementary Material 3.** Scatter plot representing linear regression of the four-resilience indicator (x-axis) on the two α-diversity measure (y-axis): of and Shannon and the inverse Simpson, within breed (DR: Duroc, LW: Large White and LR: Landrance). The four resilience indicator were Lag of one day of residual (Lag1), natural logarithm of residual variance (LnVar) area under the curve for periods with largest consecutive negative errors (MaxArea), sum of residual’s local minima (SumMin). Coefficient of determination of this (R) and p-values (p) of each regression were reported in each plot. **Supplementary Material 4.** Barplot illustrating the absolute value of the log fold change (LFC) abundances for the ten most significantly abundant KEGG pathways, for the indicators of natural logarithm of residual variance (LnVar) and area under the curve for periods with consecutive negative errors (MaxArea). KEGG pathways, with positive LFC are represented by yellow bars, while those with negative LFC are represented by light-blue bars. **Supplementary Material 5.** PERMANOVA analysis for the effect each of the four resilience phenotypes expressed in class on microbial composition. The four phenotype was lag of one day of residual (Lag1), natural logarithm of residual variance (LnVar), area under the curve for periods with the largest consecutive negative errors (MaxArea), and sum of residual's local minima (SumMin). **Supplementary Material 6.** Inverse Simpson alpha diversity in class was assessed for the four resilience indicator were Lag of one day of residual (A), natural logarithm of residual variance (B) area under the curve for periods with largest consecutive negative errors (c), sum of residual’s local minima (d).The x-axis represents the resilience classes: Lower (L), Medium (M) as the control group, and Higher (H). Above the plot, the p-values of the Kolmogorov-Smirnov test between the classes are reported above the box-plot. **Supplementary Material 7.** Heatmap illustrating the absolute value of the log fold change (LFC) abundances for the significantly abundant Kegg pathways, with the indicators of natural logarithm of residual variance (LnVar) and area under the curve for periods with consecutive negative errors (MaxArea). The ASVs are grouped based on the genera to which they belong. The x-axis represents the LFC when comparing the resilient animals class with control groups (lfc_L), and the higher resilient animals class with control groups (lfc_H). **Supplementary Material 8.**  A two-dimensional Partial Least Square Discriminant Analisis (PLS-DA) score plot was constructed using three classes of resilience (Lower (L) in yellow, Medium (M) in light blue as the control group, and Higher (H) in grey). The plot represents the distribution of the samples based on the first two components in the model. Each point's shape changes according to the breed to which the animal belongs. PLS-DA was performed for the four indicators that are lag of one day of residual (A), natural logarithm of residual variance (B), area under the curve for periods with the largest consecutive negative errors (C), and sum of residual's local minima (D).  **Supplementary Material 9.** Description of data editing.  **Supplementary Material 10.** Linear regression analysis for the effect each of the four resilience phenotypes and Room and Breed on microbial α-diversity. Analysis was performed considering each resilience phenotype each time additionally Breed and Room. The four phenotype was lag of one day of residual (Lag1), natural logarithm of residual variance (LnVar), area under the curve for periods with the largest consecutive negative errors (MaxArea), and sum of residual's local minima (SumMin).**Supplementary Material 11.** Number of animals(n)  per each resilience traits, number of animals per each class are equal among resilience indicator.

** Additional file 2.**

** Additional file 3.**

## Data Availability

The 16 S rRNA gene sequence reads for all fecal samples are available under the NCBI repository as a BioProject with accession number PRJNA747026. The resilience indicators of the host animals are readily available from the authors.

## Abbreviations

ASV: Amplicon sequence variants
DR: Duroc
LR: Landrace
LW: Large White
DA: Differential abundance analysis
LFC: Log Fold Change
H: Highly resilient group
L: Lowly resilient group
M: Middle group
m^2^: Microbiability
HPD_95_: 95% posterior density intervals
PLS-DA: Partial Least Squares - Discriminant Analysis (PLS-DA)
ROC: Receiver Operating Characteristic
SCFAs: Short-chain fatty acids
Gly: Glycine
FIRE: Feed Intake Recording Equipment
FCD: Feed Consumption Data
AUC: Area under the curve
