# Supplementary material for: A first characterization of the microbiota-resilience link in swine
Source: Microbiome. 2024 Mar 15;12:53. doi: 10.1186/s40168-024-01771-7 (PMC10941389; doi:10.1186/s40168-024-01771-7)
Supplement: Supplementary file 2 — Additional file 1: Supplementary Material 1. Tuckey Test of Breed effect resulting from the mixed models analysis for the four different resilience indicators. Supplementary Material 2. PERMANOVA analysis for the effect each of the systematic effect of the Breed and Room on the host microbiota composition. Supplementary Material 3. Scatter plot representing linear regression of the four-resilience indicator (x-axis) on the two α-diversity measure (y-axis): of and Shannon and the inverse Simpson, within breed (DR: Duroc, LW: Large White and LR: Landrance). The four resilience indicator were Lag of one day of residual (Lag1), natural logarithm of residual variance (LnVar) area under the curve for periods with largest consecutive negative errors (MaxArea), sum of residual’s local minima (SumMin). Coefficient of determination of this (R) and p-values (p) of each regression were reported in each plot. Supplementary Material 4. Barplot illustrating the absolute value of the log fold change (LFC) abundances for the ten most significantly abundant KEGG pathways, for the indicators of natural logarithm of residual variance (LnVar) and area under the curve for periods with consecutive negative errors (MaxArea). KEGG pathways, with positive LFC are represented by yellow bars, while those with negative LFC are represented by light-blue bars. Supplementary Material 5. PERMANOVA analysis for the effect each of the four resilience phenotypes expressed in class on microbial composition. The four phenotype was lag of one day of residual (Lag1), natural logarithm of residual variance (LnVar), area under the curve for periods with the largest consecutive negative errors (MaxArea), and sum of residual's local minima (SumMin). Supplementary Material 6. Inverse Simpson alpha diversity in class was assessed for the four resilience indicator were Lag of one day of residual (A), natural logarithm of residual variance (B) area under the curve for periods with largest consecutive negative err [file 40168_2024_1771_MOESM1_ESM.docx]

**Supplementary Material**

**Supplementary Material 1.** Tuckey Test of Breed effect resulting from the mixed models analysis for the four different resilience indicators.

| **Traits** | **Breed Contrast** | **Estimate** | **SE** | **t.ratio** | **p.value** |
| --- | --- | --- | --- | --- | --- |
| **Lag1** | DR - LR | -0.015 | 0.021 | -0.732 | 0.746 |
|  | DR - LW | -0.015 | 0.021 | -0.722 | 0.752 |
|  | LR - LW | 0.000 | 0.021 | 0.016 | 1.000 |
| **LnVar** | DR - LR | 0.215 | 0.231 | 0.929 | 0.626 |
|  | DR - LW | 0.176 | 0.230 | 0.765 | 0.726 |
|  | LR - LW | -0.038 | 0.233 | -0.165 | 0.985 |
| **MaxArea** | DR - LR | -0.011 | 0.161 | -0.067 | 0.998 |
|  | DR - LW | -0.089 | 0.160 | -0.558 | 0.843 |
|  | LR - LW | -0.078 | 0.162 | -0.483 | 0.880 |
| **SumMin** | DR - LR | 0.040 | 0.194 | 0.208 | 0.977 |
|  | DR - LW | -0.170 | 0.192 | -0.885 | 0.652 |
|  | LR - LW | -0.210 | 0.195 | -1.077 | 0.532 |

**Supplementary Material 2:** PERMANOVA analysis for the effect each of the systematic effect of the Breed and Room on the host microbiota composition

| Effect | DF | SumOfSqs | Pr(>F) | % |
| --- | --- | --- | --- | --- |
| Breed | 2 | 50359 | 0.000999 | 9.25 |
| Room | 7 | 25601 | 0.000999 | 4.75 |

*; SumSq the total variation between the group means and the overall mean, F test Pr(>F) p value of the F statistic, % percentage of total variance explained by microbiota composition*

**Supplementary Material 3** Scatter plot representing linear regression of the four-resilience indicator (x-axis) on the two α-diversity measure (y-axis): of and Shannon and the inverse Simpson, within breed (DR: Duroc, LW: Large White and LR: Landrance). The four resilience indicator were Lag of one day of residual (Lag1), natural logarithm of residual variance (LnVar) area under the curve for periods with largest consecutive negative errors (MaxArea), sum of residual’s local minima (SumMin). Coefficient of determination of this (R) and p-values (p) of each regression were reported in each plot.

**
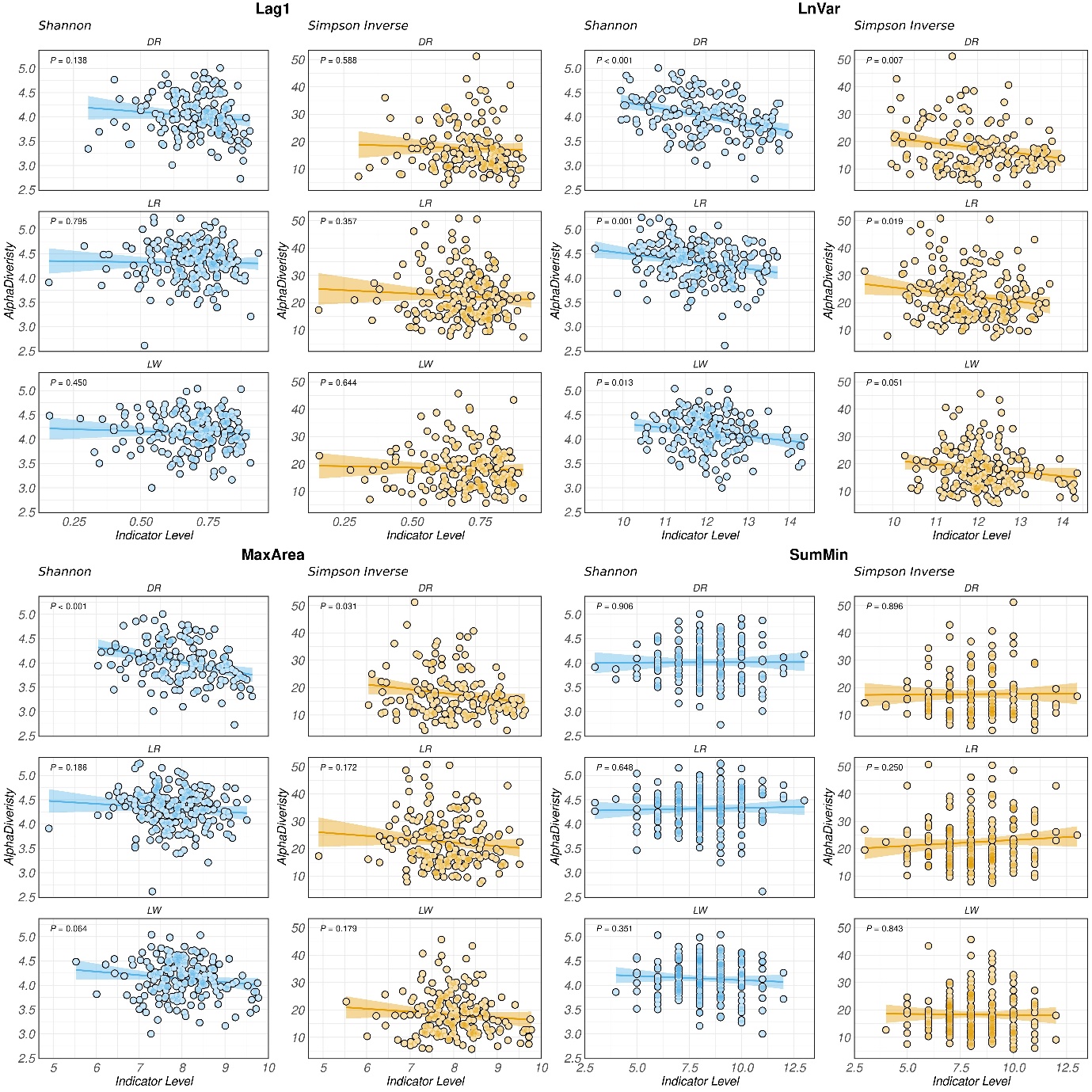
**

**Supplementary Material 4.** Barplot illustrating the absolute value of the log fold change (LFC) abundances for the ten most significantly abundant KEGG pathways, for the indicators of natural logarithm of residual variance (LnVar) and area under the curve for periods with consecutive negative errors (MaxArea). KEGG pathways, with positive LFC are represented by yellow bars, while those with negative LFC are represented by light-blue bars.

**
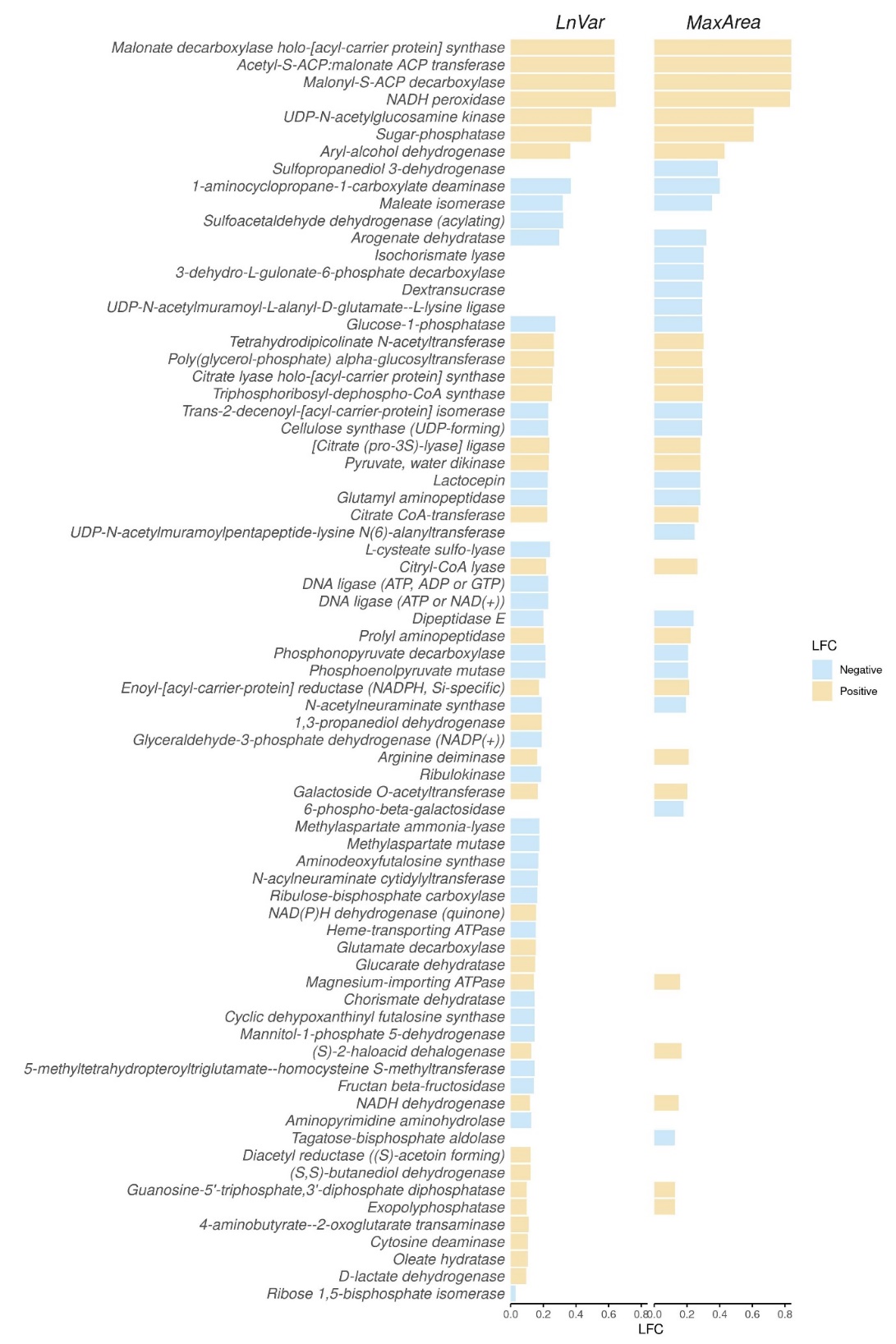
**

**Supplementary Material 5.** PERMANOVA analysis for the effect each of the four resilience phenotypes expressed in class on microbial composition. The four phenotype was lag of one day of residual (Lag1), natural logarithm of residual variance (LnVar), area under the curve for periods with the largest consecutive negative errors (MaxArea), and sum of residual's local minima (SumMin).

| **Indicator** | **SumSq** | **F** | **Pr(>F)** |
| --- | --- | --- | --- |
| Lag1 | 2798 | 1.4636 | 0.027 |
| LnVar | 3523 | 1.8317 | 0.002 |
| MaxArea | 2852 | 1.5293 | 0.015 |
| SumMin | 2301 | 1.2156 | 0.1388 |

**Supplementary Material 6.** Inverse Simpson alpha diversity in class was assessed for the four resilience indicator were Lag of one day of residual (A), natural logarithm of residual variance (B) area under the curve for periods with largest consecutive negative errors (c), sum of residual’s local minima (d).The x-axis represents the resilience classes: Lower (L), Medium (M) as the control group, and Higher (H). Above the plot, the p-values of the Kolmogorov-Smirnov test between the classes are reported above the box-plot

**
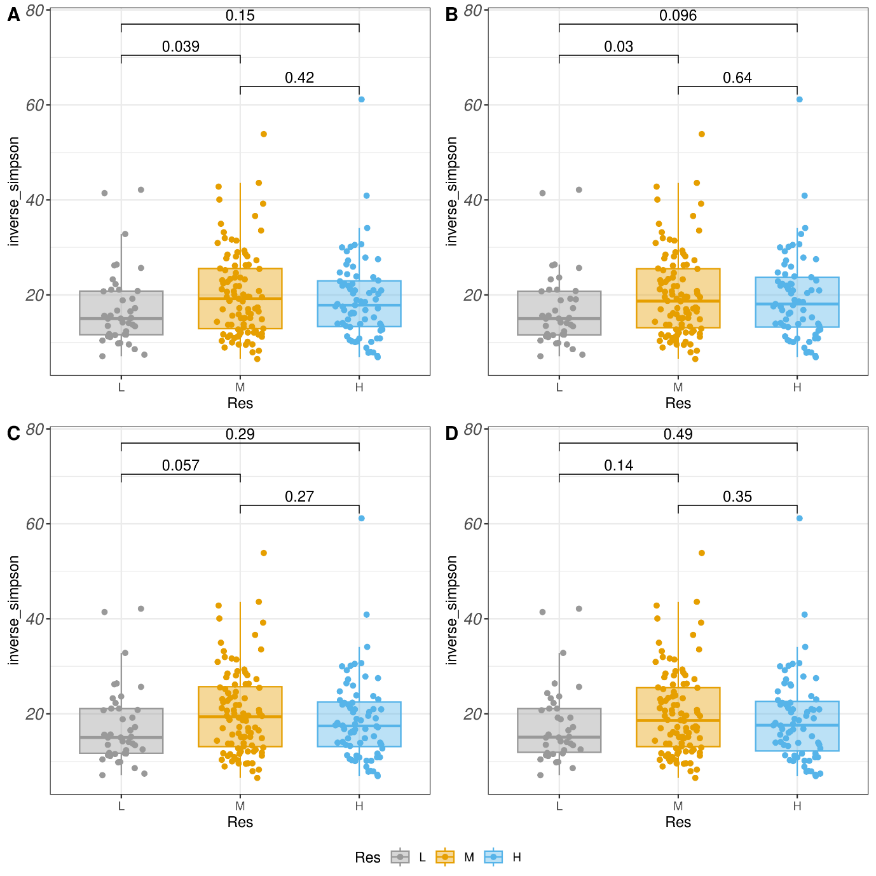
**

**Supplementary Material 7.** Heatmap illustrating the absolute value of the log fold change (LFC) abundances for the significantly abundant Kegg pathways, with the indicators of natural logarithm of residual variance (LnVar) and area under the curve for periods with consecutive negative errors (MaxArea). The ASVs are grouped based on the genera to which they belong. The x-axis represents the LFC when comparing the resilient animals class with control groups (lfc_L), and the higher resilient animals class with control groups (lfc_H).


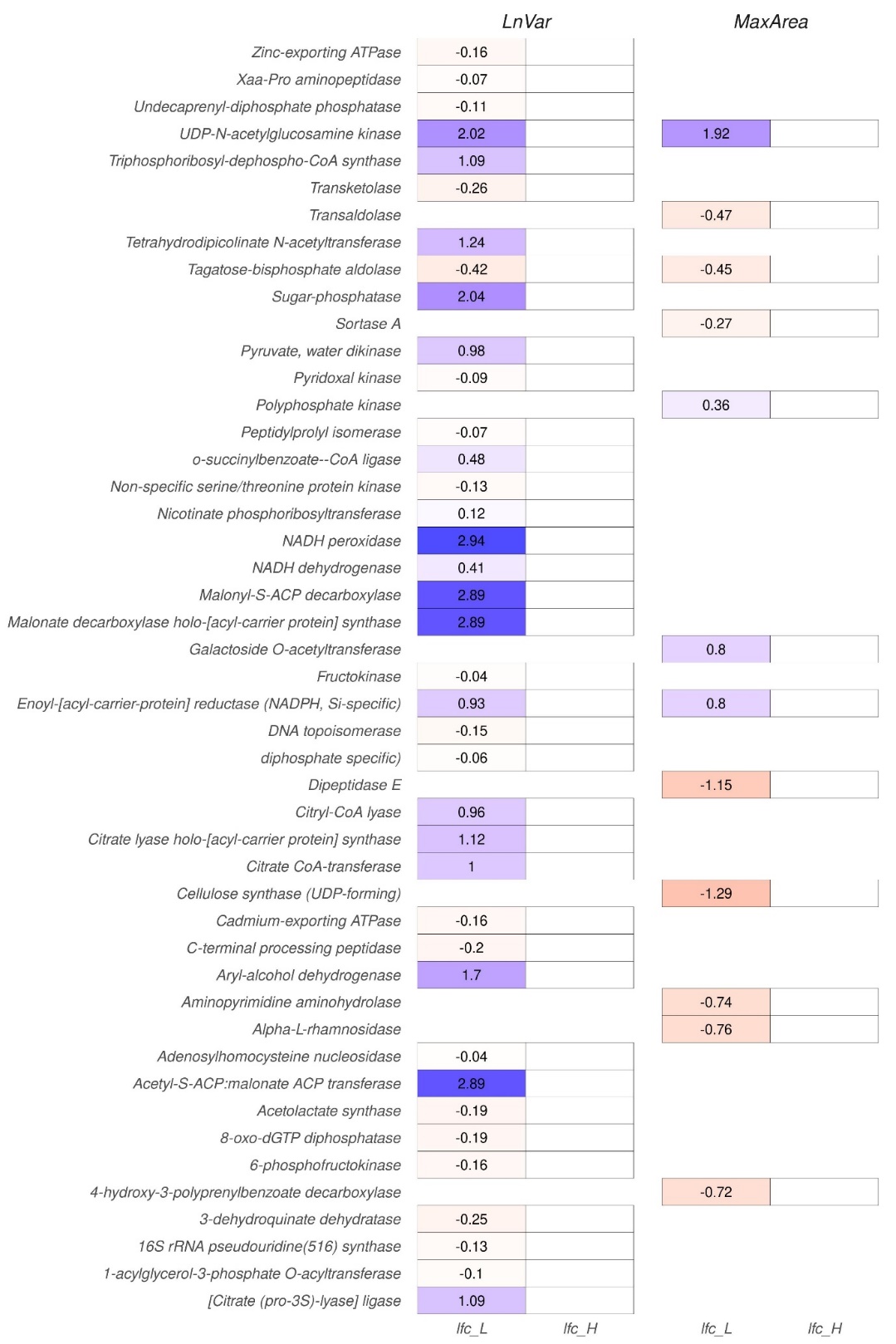


**Supplementary Material 8:**  A two-dimensional Partial Least Square Discriminant Analisis (PLS-DA) score plot was constructed using three classes of resilience (Lower (L) in yellow, Medium (M) in light blue as the control group, and Higher (H) in grey). The plot represents the distribution of the samples based on the first two components in the model. Each point's shape changes according to the breed to which the animal belongs. PLS-DA was performed for the four indicators that are lag of one day of residual (A), natural logarithm of residual variance (B), area under the curve for periods with the largest consecutive negative errors (C), and sum of residual's local minima (D).


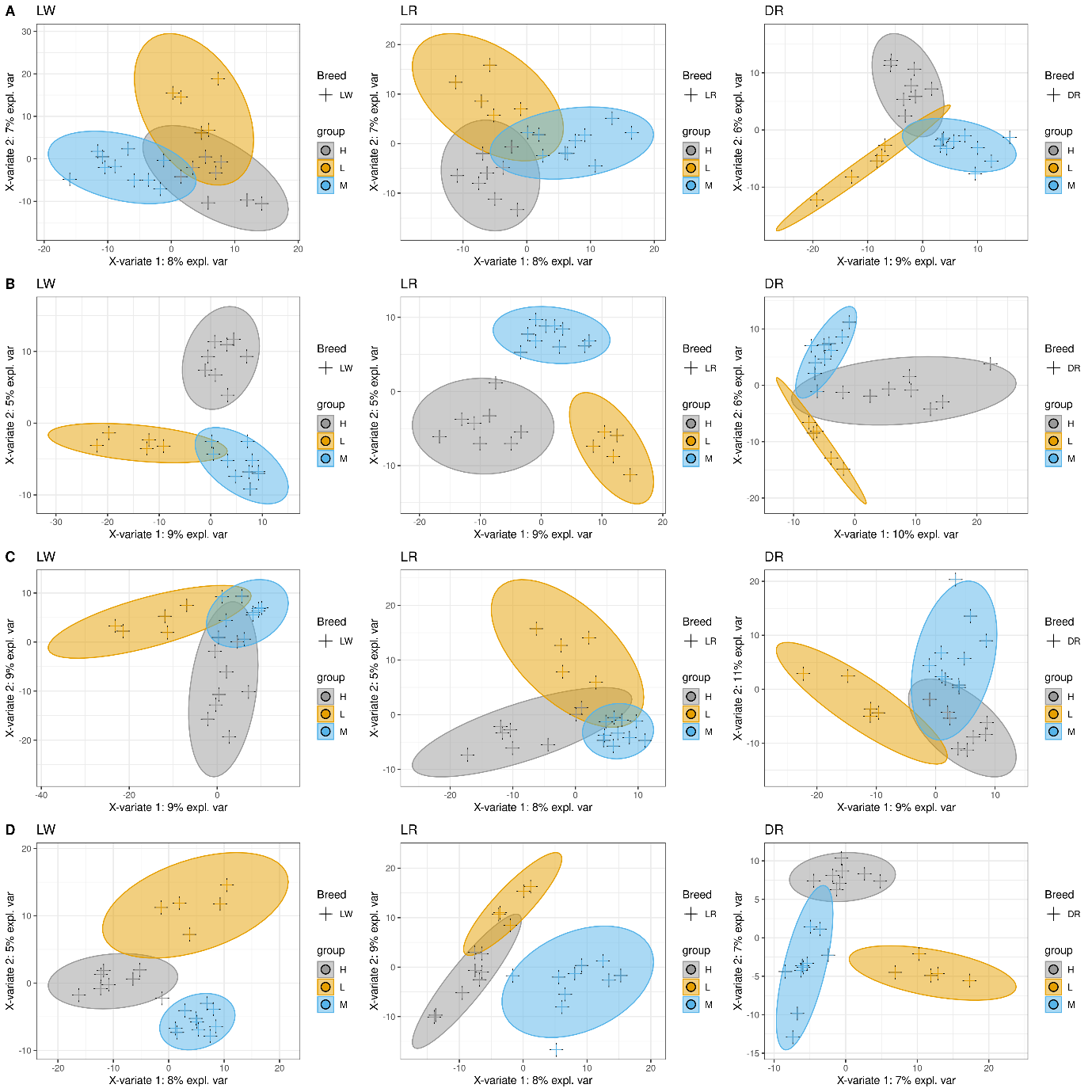


**Supplementary Material 9.** Description of data editing

An initial quality control for the single visit was performed by adapting the line guidance proposed in

Caesy (2005), to our data, in short:

1. Visits with consumption ≥ 2000g and occupation time ≥ 3600s were removed.
2. Visits with consumption ≥ 0g and ≤ 50g and feeding rate (consumption/occupation) ≥ 500(g/min) were removed.
3. Visit with consumption greater than 50g and feeding rate ≥ 170(g/s) removed.
4. Visit with feeding rate equal to zero if occupancy ≥ 500(g/s) removed.
5. Visit with feeding rate ≤ 2(g/s) were removed.
6. Visit with head and forward weight (defined bellow) difference of feed consumption ≤ -0.1 and ≥ mean + 3sd were removed
7. Visit with head and forward time (defined bellow) difference 0 were removed .

*Leading weight*: difference is the entrance weight of following visit minus exit weight of present visit.

*Following weight:* difference is entrance weight of present visit minus exit weight of preceding visit

*Leading time:* difference is the entrance time of following visit minus exit time of present visit;

*Following time:* difference entrance time of present visit minus exit time of preceding visit .

After that, a total 444,867 visit records remain ( 728 animal).Then, for each animal and for each day, we calculated the Daily feed consumption; sum of daily feed consumption for each animal (FCD).

**Supplementary Material 10.** Linear regression analysis for the effect each of the four resilience phenotypes and Room and Breed on microbial α-diversity. Analysis was performed considering each resilience phenotype each time additionally Breed and Room. The four phenotype was lag of one day of residual (Lag1), natural logarithm of residual variance (LnVar), area under the curve for periods with the largest consecutive negative errors (MaxArea), and sum of residual's local minima (SumMin).

|  | **Effect** | **Df** | **SumSq** | **MeanSq** | **F value** | **Pr(>F)** |
| --- | --- | --- | --- | --- | --- | --- |
| Shannon |  |  |  |  |  |  |
|  | Room | 7 | 15.630 | 2.2328 | 16.903 | <0.001 |
|  | Breed | 2 | 6.987 | 3.493 | 26.445 | <0.001 |
|  | Lag1 | 1 | 0.049 | 0.049 | 0.362 | 0.548 |
|  |  |  |  |  |  |  |
|  | Room | 7 | 15.630 | 2.233 | 16.903 | <0.001 |
|  | Breed | 2 | 6.987 | 3.493 | 26.445 | <0.001 |
|  | LnVar | 1 | 1.566 | 1.566 | 11.858 | <0.001 |
|  |  |  |  |  |  |  |
|  | Room | 7 | 15.630 | 2.2328 | 16.903 | <0.001 |
|  | Breed | 2 | 6.987 | 3.4933 | 26.445 | <0.001 |
|  | MaxArea | 1 | 0.304 | 0.304 | 3.028 | 0.071 |
|  |  |  |  |  |  |  |
|  | Room | 7 | 15.630 | 2.233 | 16.903 | <0.001 |
|  | Breed | 2 | 6.987 | 3.493 | 26.445 | <0.001 |
|  | SumMin | 1 | 0.203 | 0.203 | 1.503 | 0.220 |
| Inverse Shannon |  |  |  |  |  |  |
|  | Room | 7 | 5350 | 764.260 | 12.022 | <0.001 |
|  | Breed | 2 | 1972 | 985.960 | 15.509 | <0.001 |
|  | Lag1 | 1 | 2.000 | 2.000 | 0.0293 | 0.842 |
|  |  |  |  |  |  |  |
|  | Room | 7 | 5350 | 764.260 | 12.022 | <0.001 |
|  | Breed | 2 | 1972 | 985.960 | 15.509 | <0.001 |
|  | LnVar | 1 | 266.000 | 266.000 | 3.584 | 0.052 |
|  |  |  |  |  |  |  |
|  | Room | 7 | 5350.000 | 764.260 | 12.022 | <0.001 |
|  | Breed | 2 | 1972 | 985.960 | 15.509 | <0.001 |
|  | MaxArea | 1 | 10.000 | 10.000 | 1.641 | 0.685 |
|  |  |  |  |  |  |  |
|  | Room | 7 | 5350 | 764.260 | 12.022 | <0.001 |
|  | Breed | 2 | 1972 | 985.960 | 15.509 | <0.001 |
|  | SumMin | 1 | 0.203 | 0.203 | 1.5031 | 0.220 |

*SumSq the total variation between the group means and the overall mean, F test Pr(>F) p value of the F statistic*

**Supplementary Material 11.** Number of animals(n) per each resilience traits, number of animals per each class are equal among resilience indicator

| **Class** | **n** |
| --- | --- |
| H | 24 |
| L | 15 |
| M | 30 |
